# High Photosynthetic Rates in a *Solanum pennellii* Chromosome 2 QTL Is Explained by Biochemical and Photochemical Changes

**DOI:** 10.3389/fpls.2020.00794

**Published:** 2020-06-12

**Authors:** Jaciara Lana-Costa, Franklin Magnum de Oliveira Silva, Willian Batista-Silva, Diego Costa Carolino, Renato Lima Senra, David B. Medeiros, Samuel Cordeiro Vitor Martins, Jorge Gago, Wagner L. Araújo, Adriano Nunes-Nesi

**Affiliations:** ^1^Departamento de Biologia Vegetal, Universidade Federal de Viçosa, Viçosa, Brazil; ^2^Departamento de Bioquímica Aplicada, Universidade Federal de Viçosa, Viçosa, Brazil; ^3^Departament de Biologia, Institute of Agro-Environmental Research and Water Economy – INAGEA, Universitat de les Illes Balears, Palma, Spain

**Keywords:** photosynthesis, biochemical limitations, metabolism, tomato, growth

## Abstract

Enhanced photosynthesis is strictly associated with to productivity and it can be accomplished by genetic approaches through identification of genetic variation. By using a *Solanum pennellii* introgression lines (ILs) population, it was previously verified that, under normal (CO_2_), IL 2–5 and 2–6 display increased photosynthetic rates by up to 20% in comparison with their parental background (M82). However, the physiological mechanisms involved in the enhanced CO_2_ assimilation exhibited by these lines remained unknown, precluding their use for further biotechnological applications. Thereby, here we attempted to uncover the physiological factors involved in the upregulation of photosynthesis in ILs 2–5 and 2–6 under normal (CO_2_) as well as under elevated (CO_2_). The results provide evidence for increased biochemical capacity (higher maximum carboxylation velocity and maximum electron transport rate) in plants from IL 2–5 and 2–6, whereas the diffusive components (stomatal and mesophyll conductances) were unaltered in these ILs in comparison to M82. Our analyses revealed that the higher photosynthetic rate observed in these ILs was associated with higher levels of starch as well as total protein levels, specially increased RuBisCO content. Further analyses performed in plants under high (CO_2_) confirmed that biochemical properties are involved in genetic variation on chromosome 2 related to enhanced photosynthesis.

## Introduction

Photosynthesis (*A*) is the main driving force for plant growth and biomass production having a central position for breeders seeking to increase crop yield (Evans, 2013; Nunes-Nesi et al., 2016; Nuccio et al., 2017; Batista-Silva et al., 2020; Flexas and Carriquí, 2020). The improvement of photosynthetic performance under optimal and suboptimal conditions can be accomplished by genetic approaches through identification of genetic variation, as well as selection of accessions exhibiting higher photosynthetic rates. Moreover, understanding the mechanisms involved in the photosynthesis associated traits are required for improving the photosynthetic efficiency in crop species.

The photosynthetic efficiency can be enhanced, among others, by improving light capture through increasing pigment contents and by ameliorating light energy conversion (Ort et al., 2015). Furthermore, *A* can be improved by changes in stomatal properties (Rebetzke et al., 2013), ribulose 1,5 bisphosphate carboxilase oxigenase (RuBisCO) kinetic attributes (Parry et al., 2013), and also by modifying expression of others enzymes involved in carbon reduction reactions (Miyagawa et al., 2001; Lefebvre et al., 2005; Driever et al., 2017).

Alterations in *A* may be associated with diffusional limitations as those derived from changes in stomatal (*g*_s_) and mesophyll (*g*_m_) conductances which are dependent on leaf anatomical and physiological properties and influenced by environmental cues (Flexas et al., 2007, 2012; Vrabl et al., 2009; Galmés et al., 2011). For instance, high levels of CO_2_ at the substomatal cavities (*C*_i_) tend to reduce *g*_s_ in leaves by an intrinsic property of guard cells (Mott, 1988; Ainsworth and Rogers, 2007). Remarkably, at lower *C*_i_, the maximum carboxylation velocity (*V*_cmax_) limits photosynthesis. On the other hand, when *C*_i_ is higher, CO_2_ assimilation is limited by maximum electron transport rate (*J*_max_), or the regeneration of triose phosphate (Farquhar and Sharkey, 1982). Moreover, enrichment in the availability of atmospheric (CO_2_) augments RuBisCO carboxylase activity leading to reduced photorespiratory process, improving carbon gains (Long et al., 2004; Peterhansel et al., 2010).

Natural genetic variation in diffusional and biochemical traits related to photosynthetic capacity have been verified in different plant species (Geber and Dawson, 1997; Galmés et al., 2014; Singh et al., 2014). For example, Mediterranean accessions of tomato (*S. lycopersicum*) cultivated under drought stress demonstrated higher transpiration efficiency provided by enchanced *g*_m_/*g*_s_ ratio (Galmés et al., 2011). Furthermore, altered photosynthetic capacity can also be explained by components related to CO_2_ assimilation capacity namely *V*_cmax_ and *J*_max_ (Dreyer et al., 2001; Driever et al., 2014; Walker et al., 2014). Thus, selection of accessions with higher *J*_max_ contributing to ribulose 1,5 bisphosphate (RuBP) regeneration has been proposed as a trait for plant breeding (Driever et al., 2014; Simkin et al., 2017a).

The use of introgression lines (ILs) populations, an important genomic tool for Quantitative Trait Locus (QTL) mapping, has been proposed to identify candidate genes associated with improved *A* (Flood et al., 2011; Gu et al., 2012; Nunes-Nesi et al., 2016). QTL analysis allow the identification of genomic regions where detailed studies should be performed to identify genes and nucleotide changes responsible for a certain function (Asín, 2002; Gonzalez-Martinez et al., 2006; Jamil et al., 2016; de Oliveira Silva et al., 2018). In a carbon isotope composition (δ^13^C) study performed with a *Solanum pennellii* population demonstrated that variation in stomatal arrangement may improve internal CO_2_ diffusion allowing greater photosynthetic efficiency in dry environments (Xu et al., 2008; Muir et al., 2014). In addition, most of the genetic variation in tomato for *g*_s_ might be explained by differences in both width and anatomical traits of the stomatal pore (Fanourakis et al., 2015).

Recently, genomic regions involved in the regulation of photosynthesis and respiration were identified in tomato (de Oliveira Silva et al., 2018). In this study, a QTL for high *A* was identified in a unique overlapping genomic region, delimited by two ILs (IL 2–5 and IL 2–6) and named as BIN 2K. The IL 2–5 exhibited up to 20% increased *A*, 50% higher nitrate content and 21% higher starch accumulation at the end of the light period in comparison with the parental line M82. Similarly, the IL 2–6 exhibited 19% increased *A*, 22% higher *g*_s_, 42% higher levels of starch at the end of the light period, and 58% higher starch turnover in comparison with M82. In agreement, the IL 2–6 also displayed higher *g*_s_, when evaluating the variation in stomatal responsiveness to desiccation (Fanourakis et al., 2015). These two ILs were also evaluated for δ^13^C, in which IL 2–5 exhibited higher values of δ^13^C compared to M82 and IL 2–6 (−28.1, −28.4, and −28.8, respectively) indicating increased water use efficiency (*WUE*) in this line (Xu et al., 2008). Notably, the IL 2–5 was also described as a drought-tolerant line mainly through alterations in organ morphogenesis and biochemical pathways (Gong et al., 2010). In addition, the IL 2–5, and IL 2–6 have also been characterized by exihibiting higher total plant weight, number of flower, fruits and seeds than M82 (Eshed and Zamir, 1995; Semel et al., 2006). However, the fruits from these ILs have reduced size and fresh weight when compared to M82 (Eshed and Zamir, 1995; Causse et al., 2004).

In order to investigate the physiological basis for the increased *A* associated with the genomic region BIN 2K, a detailed physiological characterization of the IL 2–5, IL 2–6, and M82 was performed under atmospheric and elevated (CO_2_). Given that *A* is highly affected by environmental (CO_2_) and RuBisCO limitation is reduced by high (CO_2_; von Caemmerer and Farquhar, 1981), these ILs might exhibit higher carbon assimilation capacity under this condition. Our results demonstrate that the higher photosynthetic efficiency in the IL 2–5 and IL 2–6 was not associated with diffusive component (*g*_s_ and *g*_m_), but rather to increased photobiochemical capacity. Our findings open important avenues for enhancing our understanding of genetic variation on chromosome 2 that is involved in the regulation of photosynthetic capacity in tomato plants.

## Materials and Methods

### Plant Material and Experimental Conditions

Seeds of the ILs 2–5 and 2–6, as well as the cultivar M82 were sown in trays containing the commercial substrate Tropstrato HT, Vida Verde^®^. The plants were allocated in a greenhouse under naturally fluctuating conditions of light intensity (±600 μmol photons m^–2^ s^–1^ daily) and ambient CO_2_ (±400 μmol mol^–1^) as well as semicontrolled conditions of temperature (±28°C) and relative air humidity (±60%) during spring. After germination, seedlings were transplanted in 1.16 L pots containing the same commercial substrate, supplemented with chemical fertilizer (N:4; P_2_O_5_:14; K_2_O: 8) in the proportion of 0.5 kg of NPK to 10.0 kg of substrate. The plants were irrigated daily as needed and no restriction of root development, and consequently photosynthetic limitation, was observed at the end of the experiment when the physiological analyses and sample harvesting took place (4-week-old plants). Four plants for each genotype were grown side by side and the position of each plant was changed daily.

In a second experiment, seeds from the same genotypes were germinated and cultivated as described previoulsy with the following modifications. Pots containing plants with 10 days after transplanting (DAT) were transferred to open top chambers (OTCs) with the following specifications 1.6 m diameter and 1.8 m height. The chambers were placed in a greenhouse under naturally fluctuating conditions of light intensity (±600 μmol photons m^–2^ s^–1^ daily) and ambient CO_2_ (±400 μmol mol^–1^) as well as semicontrolled conditions of temperature (±28°C) and relative air humidity (±60%). The experiment was carried out in two OTCs. One was continuously maintained at the current ambient level of CO_2_ (∼400 μmol mol^–1^) and another continuously maintained at an elevated level of CO_2_ (∼800 μmol mol^–1^). In the chamber supplemented with elevated (CO_2_), 800 μmol mol^–1^ was the setpoint and the (CO_2_) was monitored and adjusted every day with a portable CO_2_ meter AZ-77535. CO_2_ was only injected following the photoperiod, i.e., from 06:00 a.m. to 06:00 p.m. The obtained (CO_2_) along the experiments are presented in Supplementary Figure S1. Air temperature averages (±30°C) and relative air humidity (±77%) inside the OTCs were measured throughout the experiment and did not differ significantly between the two sets of OTCs during the experiment. The two OTCs were installed at 1 m of distance between each other. The plants remained under these conditions for 3 weeks until further analysis. Four plants for each genotype were grown inside each chamber and the positions of the plants inside the chamber were daily changed.

### Growth and Fruit Analyses

Growth parameters were determined in 4-week-old plants. Total leaf area was measured using a LI-3100C Area Meter (LiCor, Lincoln, NE, United States) while specific leaf area (SLA) was measured as described previously (Hunt et al., 2002). The dry matter accumulated in leaves, stems and roots were determined as described previously (de Oliveira Silva et al., 2018). Total fruit production was determined at the end of the cultivation in plants with 60 DAT. The fruits were harvested when they presented the reddish color, characteristic of mature fruits. We determined the total number of fruits per plant, the mean fruit fresh weight per plant as well as the fruit size, by measuring the fruit length, and diameter using a digital caliper.

### Measurements of Gas Exchange Parameters and Chlorophyll *a* Fluorescence

Gas exchange and chlorophyll *a* fluorescence analyses were performed simultaneously using an open-flow infrared gas exchange analyzer system equipped with an integrated fluorescence chamber (IRGA, LI-COR Inc. LI-6400XT; NE). These analyses were realized during the light period, from 8 h to 12 h (solar time) using the 2 cm^2^ leaf chamber at 25°C, flow rate of 300 mol s^–1^, 0.5 stomatal ratio (amphistomatic leaves), and saturating light intensity of 1,000 μmol photons m^2^s^–1^. The leaf-to-air vapor pressure deficit ranged from 1.2 to 2.0 kPa and the amount of blue light was set to 10% of photosynthetic photon flux density (PPFD) to optimize stomatal aperture. The initial fluorescence (*F*_0_) was measured in illuminating dark-adapted leaves (for at least 1 h) with weak modulated measuring beams (0.03 μmol m^–2^ s^–1^). A saturating white light pulse (8,000 μmol m^–2^ s^–1^) was applied for 0.8 s to obtain the maximum fluorescence (*F*_m_), from which the variable-to-maximum Chl fluorescence ratio was then calculated: *F*_v_/*F*_m_ = [(*F*_m_–*F*_0_)/*F*_m_)]. In light-adapted leaves, the steady-state fluorescence yield was measured with the application of a saturating white light pulse (8,000 μmol m^–2^ s^–1^) to achieve the light adapted maximum fluorescence (*F*_m__’_). A far-red illumination (2 μmol m^–2^ s^–1^) was applied after turn off the actinic light to measure the light-adapted initial fluorescence (*F*_0__’_). The capture efficiency of excitation energy by open PSII reaction centers (*F*_v__’_/*F*_m__’_) was estimated following a previously described procedure (Logan et al., 2007) and the actual PSII photochemical efficiency (φPSII) was estimated as φPSII = (*F*_m__’_–*F*_s_)/*F*_m__’_ (Genty et al., 1989). Electron transport rate (ETR) was calculated as follows: ϕ*PSII*×PARi×β×α, where PARi is the incident radiation, β is the absorbed quanta partition factor between PSII/I (assumed to be 0.5), and α is the leaf absorbance. Leaf absorbance (α) was estimated by the chlorophyll content per unit area as follow: α = χ/(χ  +  76), where χ is the chlorophyll content per unit area (Evans and Poorter, 2001).

From combined measurements of fluorescence and gas exchange, we estimated the photorespiration rate (*P*_r_) according to Valentini et al. (1995). Dark respiration rate (*R*_d_) was measured by the same gas exchange system after plants being at least 2 h into the dark period.

Photosynthetic light-response curves (*A*/PPFD) were performed using atmospheric (CO_2_; *C*_a_) of 400 μmol CO_2_ mol^–1^ and the plants were exposed to a range of PPFD in the sequence: 1,000, 1,500, 1,300, 1,100, 1,000, 800, 600, 400, 300, 200, 100, 50, and 0 μmol photons m^–2^ s^–1^. Variables derived from the *A*/PPFD curves were estimated from adjustments of light response curve by the non-rectangular hyperbolic model (von Caemmerer, 2000). Measurements for CO_2_ response curves were taken at light saturation point of 1,000 μmol m^2^ s^–1^, 25°C at atmospheric CO_2_ concentration (*C*_a_) of 400 μmol mol^–1^ and once the steady state was reached, the *C*_a_ was decreased to 300, 200, 100, and 50 μmol mol^–1^. Then, *C*_a_ was increased to 400, 500, 600, 800, 1,000, 1,200, 1,400, and 1,600 μmol mol^–1^. Curves were obtained using the second terminal leaflet of the third fully expanded leaf from the apex of 4−week−old plants.

For the high (CO_2_) experiments, instantaneous gas exchange and chlorophyll *a* fluorescence analyses were performed as previously described, with minor modifications. All measurements were performed during the light period between 8 h and 12 h (solar time) under 1,000 μmol photons m^–2^ s^–1^ at the leaf level (light saturation) of PPFD, determined by *A*/PPFD curves and at 25°C. The reference (CO_2_) was set at 400 μmol mol^–1^ for plants at atmospheric (CO_2_) and 800 μmol mol^–1^ for plants under elevated (CO_2_). Instantaneous measurements were obtained using the second terminal leaflet of the third fully expanded leaf from the apex of 4−week−old plants. *R*_d_ was determined as described previously.

### Estimation of Mesophyll Conductance (*g*_m_), Maximum Rate of Carboxylation (*V*_cmax_), Maximum Rate of Electron Transport (*J*_max_), and Photosynthetic Limitations

The concentration of CO_2_ in the carboxylation sites (*C*_c_) was calculated following Harley et al. (1992) as *gm* = *A*/[*Ci*−(Γ^∗^(*ETR* + 8(*A* + *Rd*)))/(*ETR*−4(*A* + *Rd*))], and using Γ^∗^ for tomato measured previously (Hermida-Carrera et al., 2016). The *g*_m_ data were employed to convert the *A*–*C*_i_ curves into *A*–*C*_c_ curves using the following equation *C*_c_ = *C*_i_−(*A*/*g*_m_). ETR calculation using the proxy of α estimation, by the chlorophyll content empirical equation (Evans and Poorter, 2001), was additionally checked through a sensitivity analysis to ensure that the possible variability in α do not affect *g*_m_ estimates. Values for *g*_m_ were also estimated by the Ethier and Livingston (2004) method.

The parameters from *A*/*C*_c_ in plants under ambient (CO_2_), as *V*_cmax_ and *J*_max_ were calculated by fitting the mechanistic model of CO_2_ assimilation (Farquhar et al., 1980), using the *C*_c_ based on temperature of kinetic parameters of RuBisCO (*K*_c_ and *K*_o_). *V*_cmax_, *J*_max_, and *g*_m_ were normalized to 25°C using the temperature response and plug-in equations described by Sharkey et al. (2007). For the second experiment, the *V*_cmax_ single point was only calculated for plants growing under atmospheric (CO_2_; 400 μmol mol^–1^; De Kauwe et al., 2016).

Values of *A*, *g*_s_, *g*_m_, and ETR were employed to calculate the quantitative photosynthesis limitation analysis as described by Grassi and Magnani (2005) that permits the partitioning into the functional components of photosynthetic constraints related to stomatal (*l*_s_), mesophyll (*l*_m_), and leaf biochemical (*l*_b_) limitations. According to *A*–*C*_c_ curves obtained in the firt experiment, photosynthesis at the growing (CO_2_) was found at the curvi-linear part of the response, associated to the energy limited RuBP regeneration (*J*_max_). Since ETR is tightly coupled to *J*_max_, and should reflect gross photosynthesis (Genty et al., 1989; Valentini et al., 1995), calculations of *l*_b_ were confirmed using ETR as a surrogate for leaf biochemistry (Galle et al., 2009; Pons et al., 2009).

### Metabolite Analyses

Samples from fully expanded leaves were harvested in five different time points (beginning, middle and end of the light period, and as well as middle and end of the dark period) for the first experiment and two different time points (middle of the light period and end of the dark period) for the second experiment from 4−week−old plants. The material was frozen in liquid nitrogen and stored at -80°C for further analyses. Samples were homogenized and aliquots of approximately 20 mg were subjected to hot ethanol extraction as described by Cross et al. (2006). The levels of starch, glucose, fructose, and sucrose were measured as described by Fernie et al. (2001). The levels of chlorophyll (*a* and *b*) were determined exactly as previously described (Porra et al., 1989). Nitrate contents were determined as detailed by Sulpice et al. (2009) and malate and fumarate as Nunes-Nesi et al. (2007). The total protein was measured according by Bradford (1976) and the total amino acids content was determined using a colorimetric assay according to Yemm et al. (1955).

### Protein Extraction and Western Blot Analysis

Total soluble protein was extracted from 200 mg (fresh weight) of leaf tissue according to the protocol of Vasconcelos et al. (2005) and quantified using the Bradford method (Bradford, 1976). Forty micrograms of total protein was subjected to a solution containing sample buffer (125 mM Tris–HCl, pH 6.8, containing SDS 4%, 20% glycerol, 4% 2-mercaptoethanol, and 0.1% bromophenol blue). The samples were separated using 12% glycine sodium dodecyl sulfate polyacrylamide gel electrophoresis (Glycine–SDS-PAGE). The proteins were electrophoretically transferred to nitrocellulose membrane (0.45 μm; Bio-Rad) by the method of Towbin et al. (1979). The membranes were blocked for 2 h with 3% (w/v) Bovine serum albumin in Tris–buffered saline (2 M Tris–HCl, pH 7.4, containing 5 M NaCl, and 10% tween). The blots were then incubated for 2 h with specific antibodies raised in rabbit (serum diluted 1:12000 large subunit – AS03 037 code). The membranes were treated for 2 h with bridging antibodies (Anti-rabbit HRP-conjugated secondary antibodies GE Healthcare, RPN2108 code) diluted 1:10000. Large RuBisCO subunit was visualized with substrate solution (10 mg of 3,3’ -Diaminobenzidine mixed with 10 mL of Tris–buffered saline and 10 μL of H_2_O_2_). All steps were performed at room temperature. Tween-20 in Tris–buffered saline was used for washing (five times for 5 min between all treatments mentioned above) as well as for the dilution of antibodies. The bands on immunoblots were quantified by densitometry using the semi-quantitative ImageJ software (Version 1.1.4) as previously described (Tiranti et al., 2009; Brandt et al., 2018). Data were normalized by standard marker (∼50 kDa) and expressed in fold change.

### Statistical Analyses

The experiments were performed in a randomized complete block design. The data of the two experiments was submitted to analysis of variance (ANOVA). In the second experiment, two levels of the same categorical variable (CO_2_ environment) were considered. The variability of the results is expressed as the mean ± standard error (SE) of n independent biological replicates. Four biological replicates (*n* = 4) were utilized for each analysis, except for Western blot, where three biological replicate (*n* = 3) were analyzed. In order to verify the statistical difference between the genotypes, the means were compared using the Tukey test (*P* < 0.05). Pearson correlation matrices were performed to determine the correlated structure of the different parameters which were considered to be significant when *P* < 0.05. All analyses were performed using the GENES program (Cruz, 2013).

## Results

### Growth-Related Parameters

**FIGURE 1:**
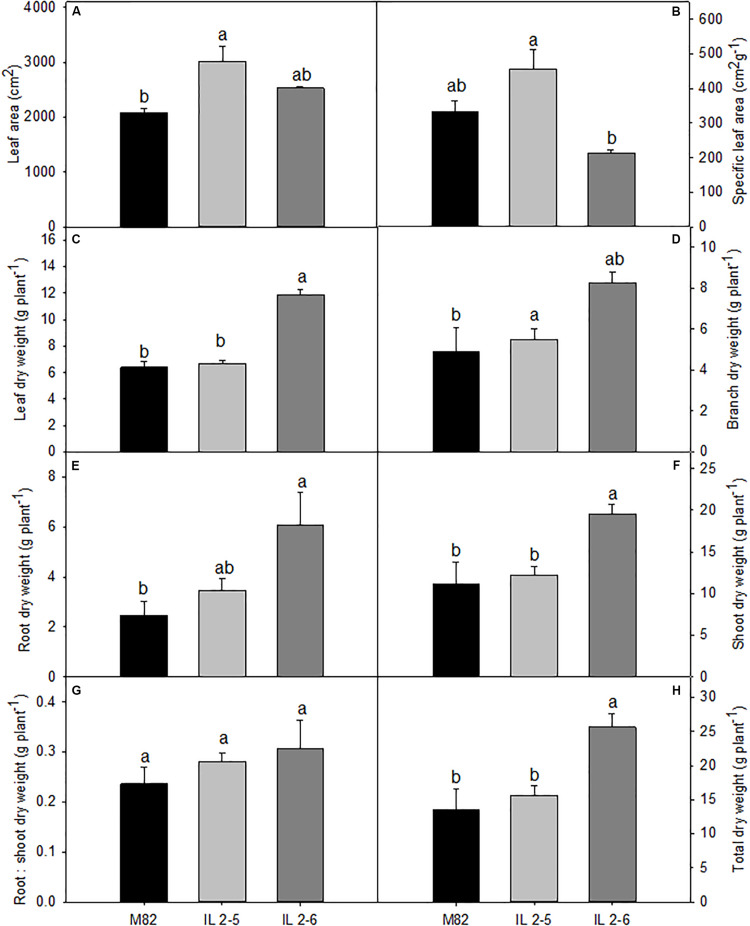
Growth phenotypes of 4–week–old plants from two ILs of *Solanum pennellii* into a genetic background of *Solanum lycopersicum* (M82) grown at 400 μmol CO_2_ mol^– 1^. Total leaf area **(A)**; Specific leaf area **(B)**; Leaf dry weight **(C)**; Branch dry weight **(D)**; Root dry weight **(E)**; Shoot dry weight **(F)**; Root: shoot dry weight ratio **(G)**; and Total dry weight **(H)**. Values are presented as means ± *SE* (*n* = 4). Different letters accompany means that differ between the genotypes (*P* < 0.05) by the Tukey test.

In order to evaluate growth related phenotypes, ILs 2–5, 2–6, and M82 plants were grown side by side in a greenhouse. Detailed examination revealed that both ILs tended to perform better than M82, whereas IL 2–5 exhibited increased total leaf area (Figure 1A), IL 2–6 displayed higher total dry weight (Figure 1H). Despite certain differences in leaf, branch, and root dry weights (Figures 1C–E), no alteration in biomass allocation was observed, as indicated by similar root:shoot ratios in all genotypes (Figure 1G).

### Photosynthesis Related Parameters

**FIGURE 2:**
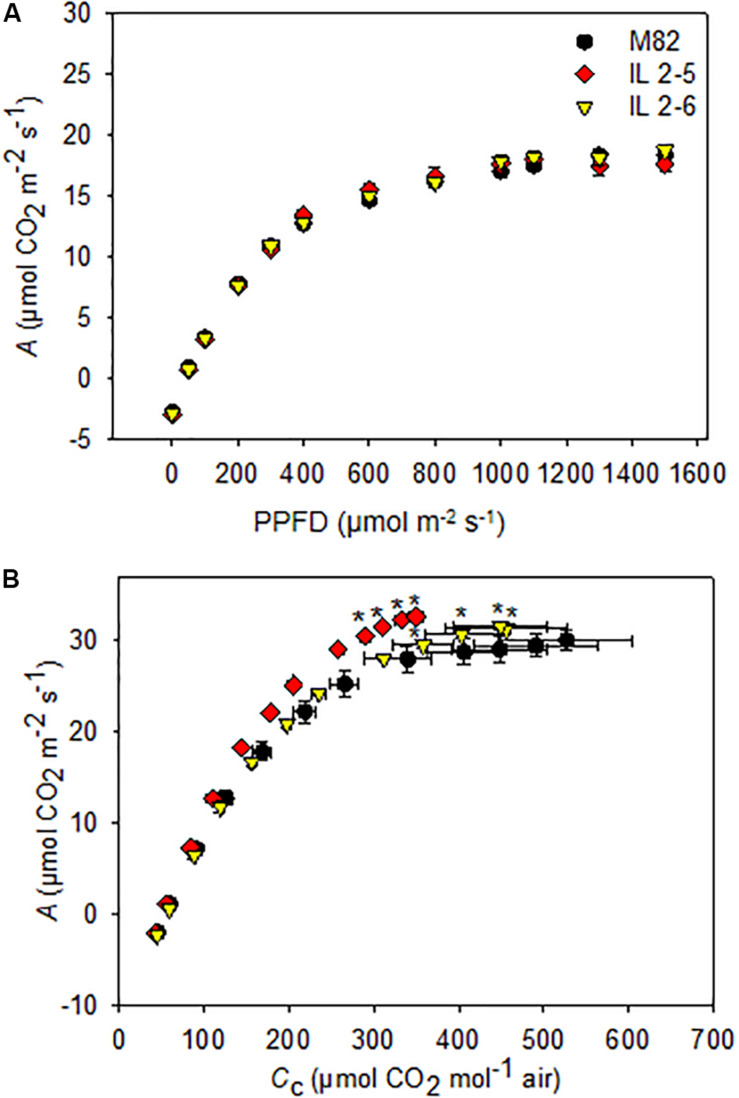
Photosynthesis response curves to photosynthetic photon flux density (*A*/PPFD) and chloroplastidic CO_2_ (*A*/*C*_c_) of 4–week–old plants from two ILs of *Solanum pennellii* into a genetic background of *Solanum lycopersicum* (M82) grown at 400 μmol CO_2_ mol^– 1^. *A*/PPFD **(A)**; *A/C*_c_ **(B)**. Values are presented as means ± *SE* (*n* = 4). Asterisks indicate differences of the ILs in relation to M82 (*P* < 0.05) according to the Tukey test.

Since the ILs 2–5 and 2–6 previously showed higher photosynthetic rate (de Oliveira Silva et al., 2018), we decided to investigate the photosynthetic capacity of these ILs by varying light intensity. We found a similar *A* response to different light intensities in all genotypes (Figure 2A), therefore no differences for light saturation and compensation points were observed (Supplementary Table S1).

We next characterized *A* in these lines under variable CO_2_ concentrations by performing *A*/*C*_c_ curve analysis. First, there were no differences in calculations on *C*_i_ basis (data not shown) but only on *C*_c_. Therefore, it was necessary to carry out a complete photosynthetic study of the calculations considering the *g*_m_, including to evaluate the photosynthetic limitations for each of the lines. Interestingly, increased photosynthetic rates were observed under specific conditions for the two ILs (Figure 2B). From *A*/*C*_c_ curves, both ILs displayed higher CO_2_ assimilation rate at the final point of the curve when compared to M82 (Figure 2B).

Concerning photosynthetic characterization of plants under atmospheric (CO_2_; Table 1), further analyses indicated that plants from the two ILs displayed similar *A*, *R*_d_, *g*_s_, and *g*_m_. On the other hand, IL 2–6 displayed increased gross photosynthesis (*A*_gross_) and both ILs higher *P*_r_, ETR and lower chloroplastic CO_2_ concentration (*C*_c_), probably due to the higher carboxylation demand, driven by the maximum RuBisCO carboxylation velocity based on *C*_c_ (*V*_cmax__*C*_c_). In addition, the *J*_max_ was higher in the ILs in comparison to M82.

**TABLE 1:** Photosynthetic characterization of 4−week−old plants from two ILs of *Solanum pennellii* into a genetic background of *Solanum lycopersicum* (M82) grown at 400 μmol CO_2_ mol^–1^.

Parameters	M82	IL 2–5	IL 2–6
*A* (μmol CO_2_ m^–2^ s^–1^)	22.80 ± 0.54**a**	21.46 ± 0.65**a**	22.67 ± 0.73**a**
*A*_gross_ (μmol CO_2_ m^–2^ s^–1^)	33.15 ± 1.33**b**	37.35 ± 0.99**ab**	37.93 ± 0.51**a**
*R*_d_ (μmol CO_2_ m^–2^ s^–1^)	2.70 ± 0.12**a**	3.09 ± 0.24**a**	3.48 ± 0.33**a**
*P*_r_ (μmol CO_2_ m^–2^ s^–1^)	7.64 ± 1.035**b**	12.80 ± 0.553**a**	11.77 ± 0.281**a**
ETR (μmol *e*^–^ m^–2^ s^–1^)	188.18 ± 13.12**b**	241.32 ± 5.522**a**	237.48 ± 2.50**a**
*g*_s_ (mol CO_2_ m^–2^ s^–1^ Pa^–1^)	0.220 ± 0.051**a**	0.205 ± 0.021**a**	0.223 ± 0.096**a**
*g*_m_*_*Harley (mol m^–2^ s^–1^ Pa^–1^)	0.188 ± 0.015**a**	0.134 ± 0.020**a**	0.172 ± 0.029**a**
*g*_m_*_*Ethier (mol m^–2^ s^–1^ Pa^–1^)	0.205 ± 0.019**a**	0.211 ± 0.027**a**	0.219 ± 0.017**a**
*C*_c_ (μmol CO_2_ mol^–1^)	175.17 ± 17.04**a**	119.522 ± 3.34**b**	127.77 ± 4.61**b**
*V*_cmax__*C*_c_ (μmol m^–2^ s^–1^)	135.68 ± 12.45**b**	225.15 ± 19.75**a**	206.25 ± 13.36**a**
*J*_max__*C*_c_ (μmol m^–2^ s^–1^)	159.50 ± 9.66**b**	228.99 ± 12.20**a**	220.28 ± 9.69**a**
*l*_s_	0.271 ± 0.007**a**	0.272 ± 0.013**a**	0.250 ± 0.013**a**
*l*_m_	0.408 ± 0.030**a**	0.484 ± 0.023**a**	0.459 ± 0.039**a**
*l*_b_	0.319 ± 0.030**a**	0.243 ± 0.015**a**	0.290 ± 0.029**a**

*These data are derived from *A*/*C*_*c*_ response curve shown in Figure 2B at ambient CO_2_ concentration (*C*_*a*_) of 400 μmol mol^–1^ setpoint. Measurements of photosynthetic carbon fixation rates were determined at saturating-light levels of 1,000 μmol m^–2^ s^–1^ in the second terminal leaflet of the third fully expanded leaf from ILs and M82 plants. Values are presented as means ± *SE* (*n* = 4). Different letters accompany means that differ between the genotypes (*P* < 0.05) by the Tukey test. *A*, net CO*_2_* assimilation rate; *A*_*gross*_, *A* + *P_*r*_* + *R*_*d*_; *R*_*d*_, dark respiration; *P*_*r*_, photorespiration; ETR, electron transport rate; *g*_*s*_, stomatal conductance; *g*_*m*_, mesophyll conductance to CO_2_; *C*_*c*_, chloroplastic CO_2_ concentration; *V*_cmax__*C*_c_, maximum carboxylation velocity based on *C*_*c*_; and *J*_*max*__*C*_*c*_, maximum capacity for electron transport rate based on *C*_*c*_; *l*_*s*_, stomatal limitation; *l*_*m*_, mesophyll limitation; and *l*_*b*_, biochemical limitation.*

### Protein and RuBisCO Content in Leaves

**FIGURE 3:**
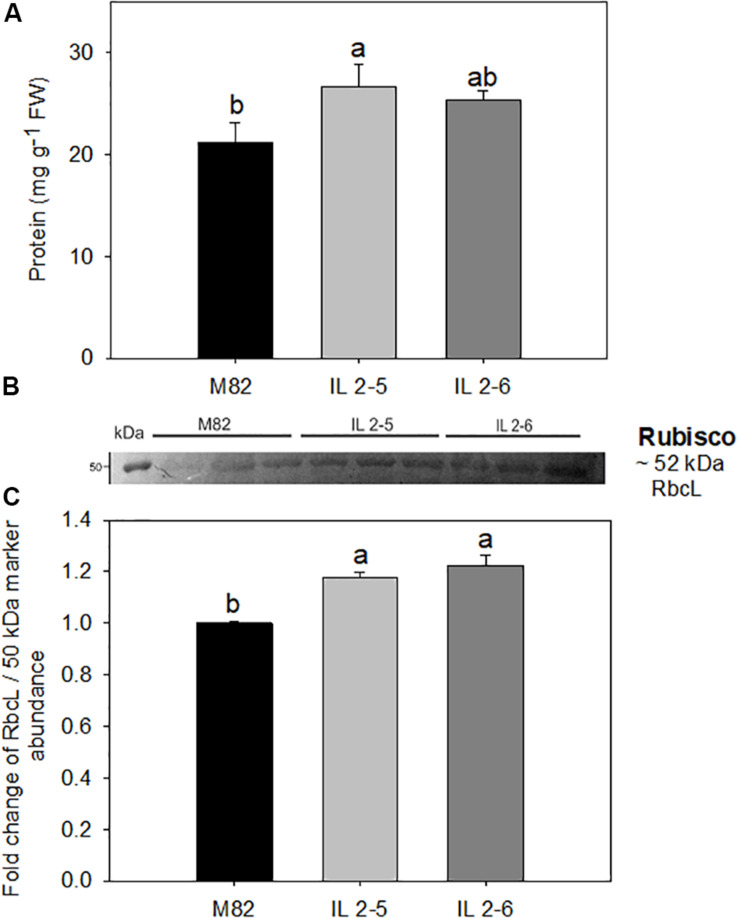
Protein content and RuBisCO large subunit (RbcL) in 4–week–old plants from two ILs of *Solanum pennellii* into a genetic background of *Solanum lycopersicum* (M82) grown at 400 μmol CO_2_ mol^– 1^. Protein **(A)**; Levels of RbcL determined by Western blot **(B)**; and quantification of western blot results **(C)**. Values for protein are presented as means ± *SE* (*n* = 4). Values for Western blot are expressed as fold change over the control M82 and presented as means ± *SE* (*n* = 3). Signal intensities were measured using ImageJ software and normalized to the amount of loaded marker (∼50 kDa). Different letters accompany means that differ between the genotypes (*P* < 0.05) by the Tukey test. FW: Fresh weight.

The total protein levels were consistently increased by up to 21% in the IL 2–5 in comparison to M82 cultivar (Figure 3A). Thus, we next analyzed the levels of RuBisCO via western blot with antibodies against its large subunit (RbcL; Figures 3B,C and Supplementary Figure S3). By quantifying the band intensity, we verified a significant increase (∼20%) in the levels of RbcL in both ILs compared to M82 (Figure 3C).

### Diurnal Pattern of Metabolite Levels in Leaves

**FIGURE 4:**
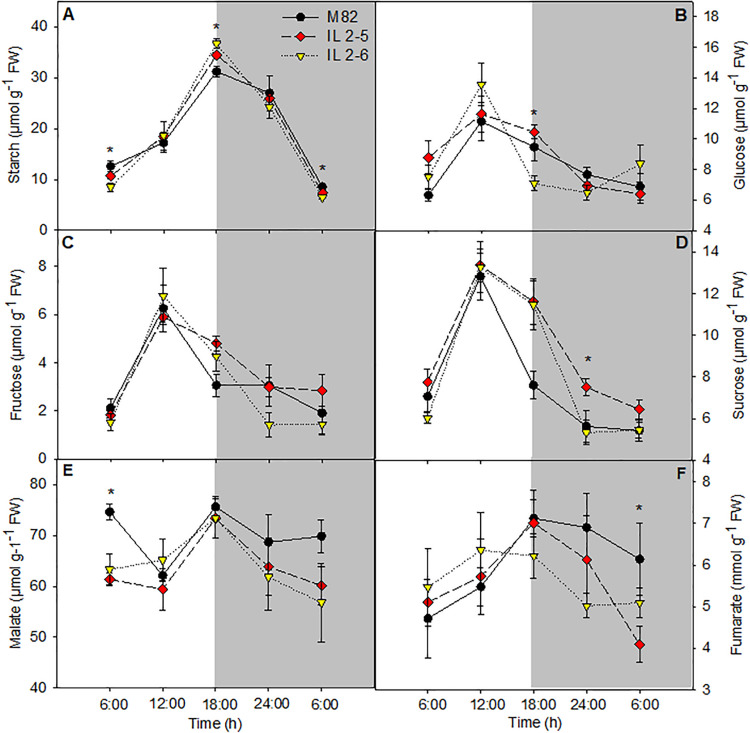
Changes in the metabolite contents involved in carbon metabolism of 4–week–old plants from two ILs of *Solanum pennellii* into a genetic background of *Solanum lycopersicum* (M82) grown at 400 μmol CO_2_ mol^– 1^. Starch **(A)**; Glucose **(B)**; Fructose **(C)**; Sucrose **(D)**; Malate **(E)**; and Fumarate **(F)**. Values are presented as means ± *SE* (*n* = 4). Asterisks indicate differences of the ILs in relation to M82 (*P* < 0.05) according to the Tukey test. FW: Fresh weight.

Increased starch content in leaves at the end of the light period in the ILs 2–5 and 2–6 was observed when compared to M82 (Figure 4A). On the other hand, at the end of dark period, the starch levels were reduced in IL 2–6, indicating higher starch degradation during the night period (Figure 4A). The glucose levels were increased at the end of the light period and sucrose showed lower levels in the middle of the night in the IL 2–5 compared to M82 (Figures 4B,D). Regarding the levels of fructose, we did not observe differences among the three genotypes (Figure 4C). The levels of malate and fumarate were generally similar to those observed in leaves of M82, except for a reduction in malate levels at the beginning of the day (Figures 4E,F) and in fumarate at the end of the night. Total chlorophyll and amino acids as well as nitrate levels did not differ among genotypes (Supplementary Figures S2A,C,D).

### Fruit Related Parameters

We also evaluated whether the greater photosynthetic efficiency would reflect on total fruit production (Supplementary Figure S4). We observed that plants from IL 2–5 produced greater number of fruits with 88% more fruits than M82 plants (Supplementary Figure S4A). However, both ILs produced smaller fruits than M82 plants, which resulted in no differences in total fruit fresh weight per plant between ILs and M82 (Supplementary Figures S4B–D).

### Elevated (CO_2_) Modifies Photosynthetic Parameters as Well as Carbon and Nitrogen Metabolism in Tomato ILs

Given the increased photosynthetic capacity observed in the ILs, indicated by the higher assimilation rate at saturating (CO_2_), we comparatively analyzed plants from M82 and the two ILs growing for 3 weeks under atmospheric and elevated (CO_2_). We noticed under the OTC conditions, which are slightly different from the greenhouse conditions, such as higher air temperature and relative air humidity, a minor decrease in the photosynthetic capacity of all genotypes as they exhibited lower *A*_gross_ and ETR (Table 2). However, the ILs still displayed higher ETR, *V*_cmax_ values (Table 2), and protein content (IL 2–5; Figure 6B), confirming the superior photobiochemistry capacity of these ILs in comparison to M82 plants. Under elevated (CO_2_), an increase of 22% in assimilation rate was observed in the ILs in comparison to M82 plants (Table 2). In addition, *A*_gross_, *P*_r_, and ETR were significantly higher in the ILs at high (CO_2_; Table 2). No changes were observed for *R*_d_, *g*_s_, and *g*_m_ at high (CO_2_) among ILs and M82 plants (Table 2). Whereas stomatal limitation was increased in the IL 2–5, biochemical limitation decreased in both ILs at elevated (CO_2_; Table 2).

**TABLE 2:** Photosynthetic characterization of 4−week−old plants from two ILs of *Solanum pennellii* into a genetic background of *Solanum lycopersicum* (M82) grown at 400 and at 800 μmol CO_2_ mol^–1^.

	400 μmol CO_2_ mol^–1^			800 μmol CO_2_ mol^–1^		
		
Parameters	M82	IL 2–5	IL 2–6	M82	IL 2–5	IL 2–6
*A* (μmol CO_2_ m^–2^ s^–1^)	18.32 ± 0.610**Aa**	20.16 ± 0.787**Aa**	20.14 ± 0.622**Aa**	18.07 ± 1.06**Ba**	22.06 ± 1.552**Aa**	21.84 ± 0.711**Aa**
*A*_gross_ (μmol CO_2_ m^–2^ s^–1^)	25.29 ± 0.876**Aa**	29.10 ± 1.30**Aa**	29.36 ± 0.99**Aa**	21.83 ± 1.07**Ba**	28.604 ± 1.85**Aa**	27.18 ± 0.80**Aa**
*R*_d_ (μmol CO_2_ m^2^ s^–1^)	1.67 ± 0.101**Aa**	1.91 ± 0.181**Aa**	1.65 ± 0.080**Aa**	1.68 ± 0.140**Aa**	1.51 ± 0.275**Aa**	1.729 ± 0.058**Aa**
*P*_r_ (μmol CO_2_ m^–2^ s^–1^)	5.32 ± 0.439**Ba**	7.02 ± 0.364**Aa**	7.57 ± 0.436**Aa**	2.07 ± 0.127**Bb**	5.01 ± 0.670**Ab**	3.60 ± 0.166**Ab**
ETR (μmol *e*^–^ m^–2^ s^–1^)	139.48 ± 6.017**Ba**	170.42 ± 7.215**Aa**	173.01 ± 7.449**Aa**	98.69 ± 3.944**Bb**	151.53 ± 11.187**Aa**	135.73 ± 3.638**Ab**
*g*_s_ (mol CO_2_ m^–2^ s^–1^ Pa^–1^)	0.229 ± 0.029**Aa**	0.248 ± 0.018**Aa**	0.241 ± 0.012**Aa**	0.233 ± 0.037**Aa**	0.187 ± 0.036**Aa**	0.219 ± 0.056**Aa**
*g*_m_ (mol CO_2_ m^–2^ s^–1^ Pa^–1^)	0.170 ± 0.018**Aa**	0.171 ± 0.0301**Aa**	0.158 ± 0.008**Aa**	0.072 ± 0.006**Ab**	0.071 ± 0.007**Ab**	0.068 ± 0.007**Ab**
*C*_c_ (μmol CO_2_ mol^–1^)	194.38 ± 10.42**Ab**	165.88 ± 1.06**Ab**	159.22 ± 4.15**Ab**	402.42 ± 15.75**Aa**	234.01 ± 24.59**Ca**	303.00 ± 11.18**Ba**
*V*_cmax__*C*_c_ (μmol m^–2^ s^–1^)	111.83 ± 6.45**B**	154.78 ± 5.30**A**	160.90 ± 7.46**A**	–	–	–
*l*_s_	0.383 ± 0.030**Aa**	0.418 ± 0.038**Aa**	0.421 ± 0.013**Aa**	0.159 ± 0.038**Bb**	0.329 ± 0.047**Aa**	0.247 ± 0.056**ABb**
*l*_m_	0.386 ± 0.020**Aa**	0.401 ± 0.034**Ab**	0.408 ± 0.028**Aa**	0.525 ± 0.037**Aa**	0.525 ± 0.037**Aa**	0.489 ± 0.051**Aa**
*l*_b_	0.230 ± 0.038**Ab**	0.180 ± 0.010**Aa**	0.169 ± 0.016**Aa**	0.416 ± 0.065**Aa**	0.181 ± 0.028**Ba**	0.262 ± 0.018**Ba**

*Measurements of photosynthetic carbon fixation rates were determined at ambient and elevated CO_2_ concentration (*C*_*a*_) of 400 μmol mol^–1^ and 800 μmol mol^–1^, respectively, and at saturating-light levels of 1,000 μmol m^–2^ s^–1^ in the second terminal leaflet of the third fully expanded leaf from ILs and M82 plants. Values are presented as means ± *SE* (*n* = 4). Capital letters compare means that differ between the genotypes within a treatment (*P* < 0.05) by the Tukey test. Lowercase letters compare means that differ in a single genotype between the two treatments (*P* < 0.05) by the Tukey test. *A*, Assimilation rate*; A*_*gross*_, *A* + *P_*r*_* + *R*_*d*_*; R*_*d*_, dark respiration; *P*_r_, photorespiration; ETR, electron transport rate; *g*_*s*,_ stomatal conductance *g*_*m*_, mesophyll conductance to CO_2_ estimated according to the Harley method; *C*_*c*_, chloroplastic CO_2_ concentration; *V*_*cmax*__ *C*_*c*_, maximum carboxylation velocity single point based on *C*_*c;*_*l*_*s*_, stomatal limitation; *l*_*m*_, mesophyll limitation; and *l*_*b*_, biochemical limitation.*

**FIGURE 5:**
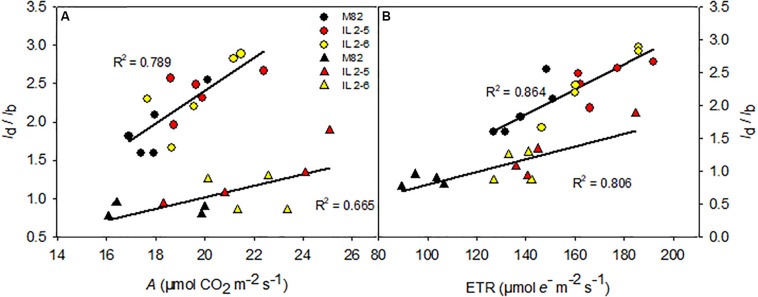
Relationships between the ratio between diffusive and biochemical photosynthetic limitations (*l*_d_*_/_l*_b_) and photosynthesis (*A*; **A**) and electron transport rate (ETR; **B**) of 4-week-old plants from two ILs of *Solanum pennellii* into a genetic background of *Solanum lycopersicum* (M82) grown at 400 and at 800 μmol CO_2_ mol^–1^. The *l_d/_l_b_* is the ratio between the diffusive limitations (*g*_s_ + *g*_m_) divided by the biochemical limitations. *g*_s_ and *g*_m,_ stomatal conductance and mesophylic conductance, respectively. Each point represents biological replicate. Circle symbol indicates genotypes cultivated under ambient (CO_2_) in the legend and triangle symbol, elevated (CO_2_). All correlations are significant at *P* < 0.005. *R*^2^: determination coefficient.

To further understand the physiological advantage provided by increased photobiochemistry in the ILs, we explored the relationship between diffusive and biochemical limitations (*l*_d_/*l*_b_) with *A* and ETR comparing ILs to M82 as well as the two growing conditions [400 and 800 ppm (CO_2_)] (Figure 5). At 400 ppm (CO_2_), as expected diffusive limitations were double than biochemicals (*l*_d_/*l*_b_ > 2) where ILs obtained higher *A* mostly due to lower biochemical limitation (thus promoted higher *l*_d_/*l*_b_ ratio than M82; Figure 5A) in comparison to M82. However, under 800 ppm (CO_2_) diffusive limitations became less important with all limitations highly balanced (*l*_d_/*l*_b_∼1), and again ILs even at high (CO_2_) showed biochemical limitations more reduced than diffusive ones (thus promoted higher *l*_d_/*l*_b_ ratio than M82) when compared to M82 (Figure 5A). The very same trend observed for the gas-exchange *A* parameter was observed for the fluorescence ETR value where ILs showed higher values than M82 at both growing conditions (Figure 5B).

**FIGURE 6:**
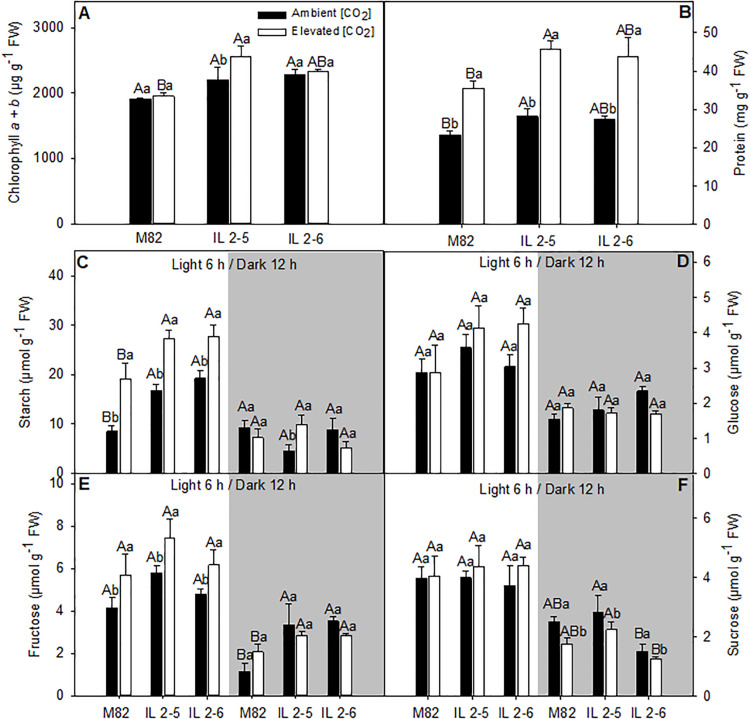
Metabolite levels of 4–week–old plants from two ILs of *Solanum pennellii* into a genetic background of *Solanum lycopersicum* (M82) grown at 400 and at 800 μmol CO_2_ mol^– 1^. Metabolite levels were measured in leaves at the middle of the light period (white sectors) and the end of dark period (gray sectors). Chlorophyll *a* + *b* **(A)**; Protein **(B)**; Starch **(C)**; Glucose **(D)**; Fructose **(E)**; and Sucrose **(F)**. Values are presented as means ± *SE* (*n* = 4). Capital letters compare means that differ between the genotypes within a treatment (*P* < 0.05) by the Tukey test. Lowercase letters compare means that differ in a single genotype between the two treatments (*P* < 0.05) by the Tukey test. FW: Fresh weight.

We next analyzed the levels of the major leaf metabolites in the ILs under elevated (CO_2_; Figure 6). In agreement with previous results suggesting higher photosynthetic efficiency of both ILs above 800 ppm (CO_2_), significant increase in the total chlorophyll (IL 2–5), and protein contents (IL 2–5) at the middle of the day, as well as fructose (both ILs) at the end of the dark period was verified (Figures 6A,B,E). Whilst sucrose at the end of the dark period increased in IL 2–5, it was reduced in IL 2–6 in ambient and elevated (CO_2_) compared to M82 (Figure 6F). On the other hand, glucose, fructose, and sucrose, total amino acids, and malate contents were unaltered at the middle of the light period in both ambients (Figures 6D–F and Supplementary Figures S5B,C). Similar to the previous observations under atmospheric (CO_2_), an increase in starch levels was observed in plants from both ILs compared to M82 at the middle of the light period (Figure 6C). Noteworthy, there was an increase in starch levels at the middle of the light period in all genotypes under elevated (CO_2_), nevertheless the ILs exhibited higher starch levels in relation to M82 (Figure 6C).

Pearson correlation analysis was applied to further comprehend how metabolic changes and structural components are driving *A* in the ILs (Supplementary Figure S6). No significant correlation was observed relating *A* and general leaf biochemical and structural parameters as total chlorophyll and protein content at both (CO_2_) treatments (Supplementary Figure S6), indicating that the higher *A* observed in both ILs is mostly driven by specific changes at the photobiochemistry level (Figure 5).

### *In silico* Expression Analysis of Genes From BIN 2K

Based on leaf expression data from a recent study (Ranjan et al., 2016), we examined the BIN 2K differentially expressed genes in leaves from IL 2–5 and IL 2–6 plants (Supplementary Table S2). We observed that a subset of 30 genes were differentialy expressed in the ILs in comparison with M82. Out of 30 genes, nine were upregulated while 19 genes were downregulated only in IL 2–5. Regarding the IL 2–6, three genes were upregulated and 11 negatively regulated. We also verified that three genes were upregulated in plants from both ILs namely an AMP-activated protein kinase (Solyc02g091530.2.1), a polypeptide inferred from GFF3 feature (Solyc02g091770.1.1), and an adiponectin receptor 2 (Solyc02g092230.2.1). Noteworthy, four genes were strongly downregulated in plants from both IL 2–5 and 2–6 including a transcription factor described as CBF_NF-Y_archaeal histone (Solyc02g090930.2.1), a multidrug resistance protein mdtK from MATE family transporter related proteins (Solyc02g091070.2.1), an epidermal patterning factor-like protein 2 (Solyc02g091910.1.1), and a formamidase-like protein (Solyc02g092530.2.1). It is important to mention, however, that caution should be taken as not to over−interpret this comparison given that the RNAseq information was obtained from an entirely independent experiment and solely for leaves.

## Discussion

Given our previous demonstration that CO_2_ assimilation rates of both ILs 2–5 and 2–6 plants were on average 20% higher compared to M82 (de Oliveira Silva et al., 2018), we decided to deeper investigate the physiological mechanisms responsible for their enhanced CO_2_ assimilation. After evaluating photosynthetic parameters, we observed that both ILs exhibited substantially higher *A* (∼22%), and ETR under high (CO_2_; Table 2), whereas no alterations were observed under ambient (CO_2_) in the lines compared to their respective M82. Regarding photosynthetic results under ambient (CO_2_), it is well established that modulations of photosynthesis mainly occur in response to environmental factors including light and temperature. In fact, the conditions of the current experiment were characterized by higher temperatures and light intensities in comparison with those previously described (de Oliveira Silva et al., 2018). Possibly, such conditions led to higher *P*_r_ (∼12 μmol CO_2_ m^–2^ s^–1^; Table 1) than those previously observed for ILs (∼8 μmol CO_2_ m^–2^ s^–1^; de Oliveira Silva et al., 2018). As a result, even with an increased *V*_cmax_ and *J*_max_ (and, consequently, *A*_gross_), increases in *A* could not be observed in the ILs at ambient (CO_2_), as the lower *C*_c_ led to higher *P*_r_ under these conditions (Table 1). Nevertheless, the two ILs exhibited increased levels of RuBisCO, while only IL 2–5 displayed significantly higher protein content under ambient (CO_2_; Figure 3). Increased protein levels can be overall associated with higher *V*_cmax_ values providing an important source of variation in carbon uptake capacity (Hikosaka, 2004) and it is in agreement with the elevated biomass production and total leaf area of the ILs (Figures 1A,H).

The increased photosynthesis in the ILs under high (CO_2_) could be explained, at least partially, by higher ETR values (Table 2). Considering that the regeneration of RuBP is determinant of the limitation of *A* under high (CO_2_; Mitchell et al., 2000), even small increments in photochemical components (e.g., ETR) may have resulted in higher photosynthetic activity in the ILs (Figure 5). The manipulation of the photosynthetic electron transport chain has been proposed as a valuable tool to enhance the carbon assimilation in plants (Simkin et al., 2017b, 2019). In agreement, transgenic Arabidopsis and tobacco plants expressing algal Cytochrome *c*_6_ gene exhibited increase in ETR, *A* as well as chlorophyll and starch contents, and further increment in biomass production (Chida et al., 2007; Yadav et al., 2018).

Close association between carbon and nitrogen metabolisms has been demonstrated in plants (Foyer et al., 1998; Araújo et al., 2008; Gauthier et al., 2010). Thus, any alteration in carbon abundance is expected to reflect in nitrogen levels (Gutschick, 1999; Nunes-Nesi et al., 2010). Among the metabolites measured in plants under elevated (CO_2_), chlorophyll and protein accumulated in the IL 2–5 as well as starch in leaves from both ILs (Figures 6A–C), which must be interpreted in light of photosynthetic data. Chlorophyll is an important part of the light-dependent reactions, and it is responsible for light harvesting during photosynthesis, therefore it has been considered an important predictor of photosynthetic potential (Croft et al., 2017; Liu et al., 2019). In its turn, the higher *A* was determining for the increased starch content, without, however, leading to the retroinhibition of the photosynthetic process (Figure 6C). In good agreement, the high protein levels of ILs could mean a higher demand for proteins involved in the metabolism and export of carbohydrates resulting from the higher photosynthetic rate.

Based both on the available leaf expression data (Chitwood et al., 2013; Ranjan et al., 2016) and in our previously published work (de Oliveira Silva et al., 2018), we verified that several genes are differentially expressed in the IL 2–5 and IL 2–6. In addition to genes mentioned here (Supplementary Table S2), three other genes appears as potential candidates involved in the responses that lead to higher photosynthetic performance observed in the ILs 2–5 and 2–6, namely the ATP−dependent Clp protease (Solyc02g091280), NADH dehydrogenase iron–sulfur protein 4 (Solyc02g092270), and beta subunit of an ATP synthase (Solyc02g091130; de Oliveira Silva et al., 2018). The proteins encoded by these genes are largely involved in energy metabolism and therefore might be involved with photosynthetic capacity. Moreover, genes enconding proteins related with photosynthesis, chlorophyll biosynthesis, and response to light stimulus were also upregulated mainly in the IL 2–5 (Ranjan et al., 2016). Noteworthy, the candidate genes for the higher photosynthetic capacity observed in plants from ILs 2–5 and 2–6 (Supplementary Table S2; and de Oliveira Silva et al., 2018) should be further investigated in more details. In this regard, introgression sublines with introgressed fragments smaller than the original set of ILs (Alseekh et al., 2013) are an alternative to reduce genetic variance and the amount of genes to be further analyzed; consequently, shortening the detection of the genes influencing photosynthetic capacity in tomato plants.

By analyzing the effects of the genetic variation on chromosome 2 of ILs 2–5 and 2–6 that modulates the upregulation of photosynthetic capacity, we have here provided novel insights into the mechanisms that govern photosynthetic capacity in tomato. Considering the higher values of *J*_max_ in ILs under ambient (CO_2_), followed by the enhanced *A* and ETR values in ILs exposed to elevated (CO_2_), it seems reasonable to assume that both ILs optimize their investment in the components involved in RuBP regeneration. Thus, genetic manipulation in these components may be an opportunity to increase photosynthesis reflecting positively on growth in a future environment with high (CO_2_; Ding et al., 2016; Driever et al., 2017; Simkin et al., 2017b), or currently in greenhouses that employ CO_2_ fertilization to improve productivity (Mortensen, 1987). Noteworthy, considering that the control of the CO_2_ assimilation in the Calvin–Benson cycle is shared among several enzymes, an increase in photosynthetic potential could be achieved combining multigene manipulation (Raines, 2003; Simkin et al., 2019). Recent studies demonstrated that increasing simultaneously the activity of different enzymes of the C3 cycle in the same plant resulted in an increased CO_2_ fixation and biomass yield dramatically (Simkin et al., 2015, 2017a). Recent challenges associated with an engineering improved photosynthesis leading to the optimized utilization of CO_2_ to maximize crop production have been discussed elsewhere (Batista-Silva et al., 2020). In this regard, the *S. pennellii* ILs population represents an opportunity to explore the large quantity of traits that affect plant survival as well as yield potential once ILs facilitate the identification of large number of genes affecting single or several plant phenotypes. Perhaps more importantly, our findings not only open up a novel fundamental research avenue, given that they identify a genomic region that lead to significant enhanced photosynthetic capacity, but also have important biotechnological implications, specifically with regard to the potential for increased yield, without increasing the need for arable land.

## Data Availability Statement

All datasets generated for this study are included in the article/Supplementary Material.

## Author Contributions

AN-N designed the research. JL-C, FO, and DC characterized the introgression lines under supervision of AN-N and WA. JL-C, WB-S, and RS performed the Western blot analyses. AN-N, FO, and JG analyzed the data. JL-C and AN-N wrote the manuscript with input from all the other authors. JG, DM, SM, and WA complemented the writing.

## Conflict of Interest

The authors declare that the research was conducted in the absence of any commercial or financial relationships that could be construed as a potential conflict of interest.

## References

[B1] Ainsworth E. A. Rogers A. (2007). The response of photosynthesis and stomatal conductance to rising [CO2]: mechanisms and environmental interactions. *Plant. Cell Environ.* 30 258–270. 10.1111/j.1365-3040.2007.01641.x 17263773

[B2] Alseekh S. Ofner I. Pleban T. Tripodi P. Di Dato F. Cammareri M. (2013). Resolution by recombination: breaking up *Solanum pennellii* introgressions. *Trends Plant Sci.* 18 536–538. 10.1016/j.tplants.2013.08.003 24029406

[B3] Araújo W. L. Nunes-Nesi A. Trenkamp S. Bunik V. I. Fernie A. R. (2008). Inhibition of 2-oxoglutarate dehydrogenase in potato tuber suggests the enzyme is limiting for respiration and confirms its importance in nitrogen assimilation. *Plant Physiol.* 148 1782–1796. 10.1104/pp.108.126219 18842826 PMC2593666

[B4] Asín M. J. (2002). Present and future of quantitative trait locus analysis in plant breeding. *Plant Breed.* 121 281–291. 10.1046/j.1439-0523.2002.730285.x

[B5] Batista-Silva W. da Fonseca-Pereira P. Oliveira Martins A. Zsögön A. Nunes-Nesi A. Araújo W. L. (2020). Engineering improved photosynthesis in the era of synthetic biology. *Plant Commun.* 1 1–17. 10.1016/j.xplc.2020.100032 PMC7747996 33367233

[B6] Bradford M. M. (1976). A rapid and sensitive method for the quantitation of microgram quantities of protein utilizing the principle of protein-dye binding. *Anal. Biochem.* 72 248–254. 10.1016/0003-2697(76)90527-90523 942051

[B7] Brandt S. Fachinger S. Tohge T. Fernie A. R. Braun H. P. Hildebrandt T. M. (2018). Extended darkness induces internal turnover of glucosinolates in *Arabidopsis thaliana* leaves. *PLoS One* 13:e0202153. 10.1371/journal.pone.0202153 30092103 PMC6084957

[B8] Causse M. Duffe P. Gomez M. C. Buret M. Damidaux R. Zamir D. (2004). A genetic map of candidate genes and QTLs involved in tomato fruit size and composition. *J. Exp. Bot.* 55 1671–1685. 10.1093/jxb/erh207 15258170

[B9] Chida H. Nakazawa A. Akazaki H. Hirano T. Suruga K. Ogawa M. (2007). Expression of the algal cytochrome c6 gene in *Arabidopsis* enhances photosynthesis and growth. *Plant Cell Physiol.* 48 948–957. 10.1093/pcp/pcm064 17548374

[B10] Chitwood D. H. Kumar R. Headland L. R. Ranjan A. Covington M. F. Ichihashi Y. (2013). A quantitative genetic basis for leaf morphology in a set of precisely defined tomato introgression lines. *Plant Cell* 25 2465–2481. 10.1105/tpc.113.112391 23872539 PMC3753377

[B11] Croft H. Chen J. M. Luo X. Bartlett P. Chen B. Staebler R. M. (2017). Leaf chlorophyll content as a proxy for leaf photosynthetic capacity. *Glob. Chang. Biol.* 23 3513–3524. 10.1111/gcb.13599 27976452

[B12] Cross J. M. von Korff M. Altmann T. Bartzetko L. Sulpice R. Gibon Y. (2006). Variation of enzyme activities and metabolite levels in 24 Arabidopsis accessions growing in carbon-limited conditions. *Plant Physiol.* 142 1574–1588. 10.1104/pp.106.086629 17085515 PMC1676042

[B13] Cruz C. D. (2013). GENES - a software package for analysis in experimental statistics and quantitative genetics. *Acta Sci.* 35 271–276. 10.4025/actasciagron.v35i3.21251

[B14] De Kauwe M. G. Lin Y.-S. Wright I. J. Medlyn B. E. Crous K. Y. Ellsworth D. S. (2016). A test of the “one-point method” for estimating maximum carboxylation capacity from field-measured, light-saturated photosynthesis. *New Phytol*. 210, 1130–1144. 10.1111/nph.13815 26719951

[B15] de Oliveira Silva F. M. Lichtenstein G. Alseekh S. Rosado-Souza L. Conte M. Suguiyama V. F. (2018). The genetic architecture of photosynthesis and plant growth-related traits in tomato. *Plant Cell Environ.* 41 327–341. 10.1111/pce.13084 29044606

[B16] Ding F. Wang M. Zhang S. Ai X. (2016). Changes in SBPase activity influence photosynthetic capacity, growth, and tolerance to chilling stress in transgenic tomato plants. *Sci. Rep.* 6:32741. 10.1038/srep32741 27586456 PMC5009361

[B17] Dreyer E. Roux X. L. Montpied P. Dauded F. Masson F. (2001). Temperature response of leaf photosynthetic capacity in seedlings from seven temperate tree species. *Tree Physiol.* 21 223–232. 10.1093/treephys/21.4.223 11276416

[B18] Driever S. M. Lawson T. Andralojc P. J. Raines C. A. Parry M. A. J. (2014). Natural variation in photosynthetic capacity, growth, and yield in 64 field-grown wheat genotypes. *J. Exp. Bot.* 65 4959–4973. 10.1093/jxb/eru253 24963002 PMC4144772

[B19] Driever S. M. Simkin A. J. Alotaibi S. Fisk S. J. Madgwick P. J. Sparks C. A. (2017). Increased sbpase activity improves photosynthesis and grain yield in wheat grown in greenhouse conditions. *Philos. Trans. R. Soc. B Biol. Sci.* 372:384. 10.1098/rstb.2016.0384 28808101 PMC5566882

[B20] Eshed Y. Zamir D. (1995). An introgression line population of Lycopersicon pennellii in the cultivated tomato enables the identification and fine mapping of yield-associated QTL. *Genetics* 141 1147–1162. 8582620 10.1093/genetics/141.3.1147 PMC1206837

[B21] Ethier G. J. Livingston N. J. (2004). On the need to incorporate sensitivity to CO2 transfer conductance into the farquhar-von caemmerer-berry leaf photosynthesis model. *Plant Cell Environ.* 27 137–153. 10.1111/j.1365-3040.2004.01140.x

[B22] Evans J. R. (2013). Improving photosynthesis. *Plant Physiol.* 162 1780–1793. 10.1104/pp.113.219006 23812345 PMC3729760

[B23] Evans J. R. Poorter H. (2001). Photosynthetic acclimation of plants to growth irradiance: the relative importance of specific leaf area and nitrogen partitioning in maximizing carbon gain. *Plant, Cell Environ.* 24 755–767. 10.1046/j.1365-3040.2001.00724.x

[B24] Fanourakis D. Giday H. Milla R. Pieruschka R. Kjaer K. H. Bolger M. (2015). Pore size regulates operating stomatal conductance, while stomatal densities drive the partitioning of conductance between leaf sides. *Ann. Bot.* 115 555–565. 10.1093/aob/mcu247 25538116 PMC4343285

[B25] Farquhar G. D. Sharkey T. D. (1982). Stomatal conductance and photosynthesis. *Annu. Rev. Plant Physiol.* 33 317–345. 10.1146/annurev.pp.33.060182.001533

[B26] Farquhar G. D. von Caemmerer S. Berry J. A. (1980). A biochemical model of photosynthetic CO2 assimilation in leaves of C3 species. *Planta* 149 78–90. 10.1007/BF00386231 24306196

[B27] Fernie A. R. Roscher A. Ratcliffe R. G. Kruger N. J. (2001). Fructose 2,6-bisphosphate activates pyrophosphate: fructose-6-phosphate 1-phosphotransferase and increases triose phosphate to hexose phosphate cycling in heterotrophic cells. *Planta* 212 250–263. 10.1007/s004250000386 11216846

[B28] Flexas J. Barbour M. M. Brendel O. Cabrera H. M. Carriquí M. Díaz-Espejo A. (2012). Mesophyll diffusion conductance to CO2: an unappreciated central player in photosynthesis. *Plant Sci.* 193-194 70–84. 10.1016/J.PLANTSCI.2012.05.009 22794920

[B29] Flexas J. Carriquí M. (2020). Photosynthesis and photosynthetic efficiencies along the terrestrial plant’s phylogeny: lessons for improving crop photosynthesis. *Plant J.* 101 964–978. 10.1111/tpj.14651 31833133

[B30] Flexas J. Diaz-Espejo A. Galmés J. Kaldenhoff R. Medrano H. Ribas-Carbo M. (2007). Rapid variations of mesophyll conductance in response to changes in CO 2 concentration around leaves. *Plant Cell Environ.* 30 1284–1298. 10.1111/j.1365-3040.2007.01700.x 17727418

[B31] Flood P. J. Harbinson J. Aarts M. G. M. (2011). Natural genetic variation in plant photosynthesis. *Trends Plant Sci.* 16 327–335. 10.1016/j.tplants.2011.02.005 21435936

[B32] Foyer C. H. Valadier M.-H. Migge A. Becker T. W. (1998). Drought-induced effects on nitrate reductase activity and mRNA and on the coordination of nitrogen and carbon metabolism in maize leaves. *Plant Physiol.* 117 283–292. 10.1104/PP.117.1.283 9576798 PMC35013

[B33] Galle A. Florez-sarasa I. Tomas M. Pou A. Medrano H. Ribas-carbo M. (2009). The role of mesophyll conductance during water stress and recovery in tobacco (*Nicotiana sylvestris*): acclimation or limitation? *Cell* 60 2379–2390. 10.1093/jxb/erp071 19321646

[B34] Galmés J. Conesa M. A. Ochogavía J. M. Perdomo J. A. Francis D. M. Ribas-Carbó M. (2011). Physiological and morphological adaptations in relation to water use efficiency in mediterranean accessions of *Solanum lycopersicum*. *Plant Cell Environ.* 34 245–260. 10.1111/j.1365-3040.2010.02239.x 20955222

[B35] Galmés J. Kapralov M. V. Andralojc P. J. Conesa M. À Keys A. J. Parry M. A. J. (2014). Expanding knowledge of the *Rubisco kinetics* variability in plant species: environmental and evolutionary trends. *Plant Cell Environ.* 37 1989–2001. 10.1111/pce.12335 24689692

[B36] Gauthier P. P. G. Bligny R. Gout E. Mahé A. Nogués S. Hodges M. (2010). In folio isotopic tracing demonstrates that nitrogen assimilation into glutamate is mostly independent from current CO2 assimilation in illuminated leaves of *Brassica napus*. *New Phytol.* 185 988–999. 10.1111/j.1469-8137.2009.03130.x 20070539

[B37] Geber M. A. Dawson T. E. (1997). Genetic variation in stomatal and biochemical limitations to photosynthesis in the annual plant, *Polygonum arenastrum*. *Oecologia* 109 535–546. 10.1007/s004420050114 28307337

[B38] Genty B. Briantais J.-M. Baker N. R. (1989). The relationship between the quantum yield of photosynthetic electron transport and quenching of chlorophyll fluorescence. *Biochim. Biophys. Acta Gen. Subj.* 990 87–92. 10.1016/S0304-4165(89)80016-80019

[B39] Gong P. Zhang J. Li H. Yang C. Zhang C. Zhang X. (2010). Transcriptional profiles of drought-responsive genes in modulating transcription signal transduction, and biochemical pathways in tomato. *J. Exp. Bot.* 61 3563–3575. 10.1093/jxb/erq167 20643807 PMC2921197

[B40] Gonzalez-Martinez S. C. Krutovsky K. V. Neale D. B. (2006). Forest-tree population genomics and adaptive evolution. *New Phytol.* 170 227–238. 10.1111/j.1469-8137.2006.01686.x 16608450

[B41] Grassi G. Magnani F. (2005). Stomatal, mesophyll conductance and biochemical limitations to photosynthesis as affected by drought and leaf ontogeny in ash and oak trees. *Plant Cell Environ.* 28 834–849. 10.1111/j.1365-3040.2005.01333.x

[B42] Gu J. Yin X. Struik P. C. Stomph T. J. Wang H. (2012). Using chromosome introgression lines to map quantitative trait loci for photosynthesis parameters in rice (*Oryza sativa* L.) leaves under drought and well-watered field conditions. *J. Exp. Bot.* 63 455–469. 10.1093/jxb/err292 21984650 PMC3245479

[B43] Gutschick V. P. (1999). Research reviews biotic and abiotic consequences of differences in leaf structure. *New Phytol.* 143 3–18. 10.1046/j.1469-8137.1999.00423.x

[B44] Harley P. C. Loreto F. Marco G. Sharkey T. D. (1992). Theoretical considerations when estimating the mesophyll conductance to CO2 flux by analysis of the response of photosynthesis to CO2. *Plant Physiol.* 98:1429. 10.1104/pp.98.4.1429 16668811 PMC1080368

[B45] Hermida-Carrera C. Kapralov M. V. Galmés J. (2016). Rubisco catalytic properties and temperature response in crops 1. *Plant Physiol.* 171 2549–2561. 10.1104/pp.16.01846 27329223 PMC4972260

[B46] Hikosaka K. (2004). Interspecific difference in the photosynthesis - nitrogen relationship: patterns, physiological causes, and ecological importance. *J. Plant Res.* 117 481–494. 10.1007/s10265-004-0174-172 15583974

[B47] Hunt R. Causton D. R. Shipley B. Askew A. P. (2002). A modern tool for classical plant growth analysis. *Ann. Bot.* 90 485–488. 10.1093/AOB/MCF214 12324272 PMC4240380

[B48] Jamil M. Ali A. Akbar K. F. Napar A. A. Gul A. (2016). *QTL Analysis in Plants: Ancient and Modern Perspectives.* Berlin: Springer International Publishing.

[B49] Lefebvre S. Lawson T. Fryer M. Zakhleniuk O. V. Lloyd J. C. Raines C. A. (2005). Increased sedoheptulose-1,7-bisphosphatase activity in transgenic tobacco plants stimulates photosynthesis and growth from an early stage in development 1. *Plant Physiol.* 138 451–460. 10.1104/pp.104.055046 15863701 PMC1104198

[B50] Liu C. Liu Y. Lu Y. Liao Y. Nie J. Yuan X. (2019). Use of a leaf chlorophyll content index to improve the prediction of above-ground biomass and productivity. *PeerJ* 6:e6240. 10.7717/peerj.6240 30648006 PMC6330949

[B51] Logan B. A. Adams W. W. Demmig-Adams B. (2007). Avoiding common pitfalls of chlorophyll fluorescence analysis under field conditions. *Funct. Plant Biol.* 34 853–859. 10.1071/FP07113 32689413

[B52] Long S. P. Ainsworth E. A. Rogers A. Ort D. R. (2004). Rising atmospheric carbon dioxide: plants FACE the future. *Annu. Rev. Plant Biol.* 55 591–628. 10.1146/annurev.arplant.55.031903.141610 15377233

[B53] Mitchell R. A. C. Theobald J. C. Lawlor D. W. (2000). Is there scope for improving balance between RuBP-regeneration and carboxylation capacities in wheat at elevated CO2? *J. Exp. Bot.* 51 391–397. 10.1093/jexbot/51.suppl_1.391 10938847

[B54] Miyagawa Y. Tamoi M. Shigeoka S. (2001). Overexpression of a cyanobacterial fructose-1,6-/sedoheptulose-1,7-bisphosphatase in tobacco enhances photosynthesis and growth. *Nat. Biotechnol.* 19 965–969. 10.1038/nbt1001-965 11581664

[B55] Mortensen L. (1987). Review: CO2 enrichment in greenhouses. *Crop Responses. Sci. Hortic.* 33 1–25. 10.1016/0304-4238(87)90028-8

[B56] Mott K. A. (1988). Do stomata respond to CO2 concentrations other than intercellular? *Plant Physiol.* 86 200–203. 10.1104/pp.86.1.200 16665866 PMC1054454

[B57] Muir C. D. Pease J. B. Moyle L. C. (2014). Quantitative genetic analysis indicates natural selection on leaf phenotypes across wild tomato species (*Solanum sect*. *lycopersicon; solanaceae)*. *Genetics* 198 1629–1643. 10.1534/genetics.114.169276 25298519 PMC4256776

[B58] Nuccio M. L. Potter L. Stiegelmeyer S. M. Curley J. Cohn J. Wittich P. E. (2017). Strategies and tools to improve crop productivity by targeting photosynthesis. *Philso. Trans. R. Soc. B* 372:20160377. 10.1098/rstb.2016.0377 28808096 PMC5566877

[B59] Nunes-Nesi A. Carrari F. Gibon Y. Sulpice R. Lytovchenko A. Fisahn J. (2007). Deficiency of mitochondrial fumarase activity in tomato plants impairs photosynthesis via an effect on stomatal function. *Plant J.* 50 1093–1106. 10.1111/j.1365-313X.2007.03115.x 17461782

[B60] Nunes-Nesi A. Fernie A. R. Stitt M. (2010). Metabolic and signaling aspects underpinning the regulation of plant carbon nitrogen interactions. *Mol. Plant* 3 973–996. 10.1093/mp/ssq049 20926550

[B61] Nunes-Nesi A. Nascimento V. D. L. De Oliveira Silva F. M. Zsögön A. Araújo W. L. Sulpice R. (2016). Natural genetic variation for morphological and molecular determinants of plant growth and yield. *J. Exp. Bot.* 67 2989–3001. 10.1093/jxb/erw124 27012286

[B62] Ort D. R. Merchant S. S. Alric J. Barkan A. Blankenship R. E. Bock R. (2015). Redesigning photosynthesis to sustainably meet global food and bioenergy demand. *PNAS* 112 8529–8536. 10.1073/pnas.1424031112 26124102 PMC4507207

[B63] Parry M. A. J. Andralojc P. J. Scales J. C. Salvucci M. E. Carmo-Silva A. E. Alonso H. (2013). Rubisco activity and regulation as targets for crop improvement. *J. Exp. Bot.* 64 717–730. 10.1093/jxb/ers336 23162118

[B64] Peterhansel C. Horst I. Niessen M. Blume C. Kebeish R. Kürkcüoglu S. (2010). Photorespiration. *Arab. B* 8:e0130. 10.1199/tab.0130 22303256 PMC3244903

[B65] Pons T. L. Flexas J. Caemmerer S. Von Evans J. R. Genty B. Ribas-carbo M. (2009). Estimating mesophyll conductance to CO2: methodology, potential errors, and recommendations. *J. Exp. Bot.* 60 2217–2234. 10.1093/jxb/erp081 19357431

[B66] Porra R. J. Thompson W. A. Kriedemann P. E. (1989). Determination of accurate extinction coefficients and simultaneous equations for assaying chlorophylls a and b extracted with four different solvents: verification of the concentration of chlorophyll standards by atomic absorption spectroscopy. *Biochim. Biophys. Acta Bioenerg.* 975 384–394. 10.1016/S0005-2728(89)80347-80340

[B67] Raines C. A. (2003). The calvin cycle revisited. *Photosynth. Res.* 75 1–10. 10.1023/A:1022421515027 16245089

[B68] Ranjan A. Budke J. M. Rowland S. D. Chitwood D. H. Kumar R. Carriedo L. (2016). eQTL regulating transcript levels associated with diverse biological processes in tomato. *Plant Physiol.* 172 328–340. 10.1104/pp.16.00289 27418589 PMC5074602

[B69] Rebetzke G. J. Rattey A. R. Farquhar G. D. Richards R. A. Condon A. G. (2013). Genomic regions for canopy temperature and their genetic association with stomatal conductance and grain yield in wheat. *Funct. Plant Biol.* 40:14. 10.1071/FP12184 32481083

[B70] Semel Y. Nissenbaum J. Menda N. Zinder M. Krieger U. Issman N. (2006). Overdominant quantitative trait loci for yield and fitness in tomato. *Proc. Natl. Acad. Sci. U.S.A.* 103 12981–12986. 10.1073/pnas.0604635103 16938842 PMC1552043

[B71] Sharkey T. D. Bernacchi C. J. Farquhar G. D. Singsaas E. L. (2007). Fitting photosynthetic carbon dioxide response curves for C 3 leaves. *Plant. Cell Environ.* 30 1035–1040. 10.1111/j.1365-3040.2007.01710.x 17661745

[B72] Simkin A. J. Lopez-Calcagno P. E. Davey P. A. Headland L. R. Lawson T. Timm S. (2017a). Simultaneous stimulation of sedoheptulose 1,7-bisphosphatase, fructose 1,6-bisphophate aldolase and the photorespiratory glycine decarboxylase-H protein increases CO2 assimilation, vegetative biomass and seed yield in *Arabidopsis*. *Plant Biotechnol. J.* 15 805–816. 10.1111/pbi.12676 27936496 PMC5466442

[B73] Simkin A. J. Mcausland L. Lawson T. Raines C. A. (2017b). Overexpression of the RieskeFeS protein increases electron transport rates and biomass yield 1 [CC-BY]. *Science* 175 134–145. 10.1104/pp.17.00622 PMC5580758 28754840

[B74] Simkin A. J. López-Calcagno P. E. Raines C. A. Lunn J. E. (2019). Feeding the world: improving photosynthetic efficiency for sustainable crop production. *J. Exp. Bot.* 70 1119–1140. 10.1093/jxb/ery445 30772919 PMC6395887

[B75] Simkin A. J. Mcausland L. Headland L. R. Lawson T. Raines C. A. (2015). Multigene manipulation of photosynthetic carbon assimilation increases CO2 fixation and biomass yield in tobacco. *J. Exp. Bot.* 66 4075–4090. 10.1093/jxb/erv204 25956882 PMC4473996

[B76] Singh J. Pandey P. James D. Chandrasekhar K. Achary V. M. M. Kaul T. (2014). Enhancing C3 photosynthesis: an outlook on feasible interventions for crop improvement. *Plant Biotechnol. J.* 12 1217–1230. 10.1111/pbi.12246 25196090

[B77] Sulpice R. Pyl E.-T. Ishihara H. Trenkamp S. Steinfath M. Witucka-Wall H. (2009). Starch as a major integrator in the regulation of plant growth. *Proc. Natl. Acad. Sci. U.S.A.* 106 10348–10353. 10.1073/pnas.0903478106 19506259 PMC2693182

[B78] Tiranti V. Viscomi C. Hildebrandt T. Di Meo I. Mineri R. Tiveron C. (2009). Loss of ETHE1, a mitochondrial dioxygenase, causes fatal sulfide toxicity in ethylmalonic encephalopathy. *Nat. Med.* 15 200–205. 10.1038/nm.1907 19136963

[B79] Towbin H. Staehelin T. Gordon J. (1979). Electrophoretic transfer of proteins from polyacrylamide gels to nitrocellulose sheets: procedure and some applications. *Proc. Natl. Acad. Sci. U.S.A.* 76 4350–4354. 10.1073/pnas.76.9.4350 388439 PMC411572

[B80] Valentini R. Epron D. de Angelis P. Matteucci G. Dreyer E. (1995). In situ estimation of net CO2 assimilation, photosynthetic electron flow and photorespiration in Turkey oak (*Q. cerris* L.) leaves: diurnal cycles under different levels of water supply. *Plant. Cell Environ.* 18 631–640. 10.1111/j.1365-3040.1995.tb00564.x

[B81] Vasconcelos ÉA. R. Nogueira F. C. S. Abreu E. F. M. Gonçalves E. F. Souza P. A. S. Campos F. A. P. (2005). Protein extraction from cowpea tissues for 2-D Gel electrophoresis and MS analysis. *Chromatographia* 62 447–450. 10.1365/s10337-005-0637-631

[B82] von Caemmerer S. (2000). *Biochemical Models Of Leaf Photosynthesis.* Clayton: CSIRO Publishing.

[B83] von Caemmerer S. Farquhar G. D. (1981). Some relationships between the biochemistry of photosynthesis and the gas exchange of leaves. *Planta* 153 376–387. 10.1007/BF00384257 24276943

[B84] Vrabl D. Vas Kova M. Hronkova M. Flexas J. Antru C. Ek J. S. (2009). Mesophyll conductance to CO2 transport estimated by two independent methods: effect of variable CO2 concentration and abscisic acid. *J. Exp. Bot.* 60 2315–2323. 10.1093/jxb/erp115 19433478

[B85] Walker A. P. Beckerman A. P. Gu L. Kattge J. Cernusak L. A. Domingues T. F. (2014). The relationship of leaf photosynthetic traits - *V* cmax and *J* max - to leaf nitrogen, leaf phosphorus, and specific leaf area: a meta-analysis and modeling study. *Ecol. Evol.* 4 3218–3235. 10.1002/ece3.1173 25473475 PMC4222209

[B86] Xu X. Martin B. Comstock J. P. Vision T. J. Tauer C. G. Zhao B. (2008). Fine mapping a QTL for carbon isotope composition in tomato. *Theor. Appl. Genet.* 117 221–233. 10.1007/s00122-008-0767-766 18542914

[B87] Yadav S. K. Khatri K. Rathore M. S. Jha B. (2018). Introgression of UfCyt c 6, a thylakoid lumen protein from a green seaweed *Ulva fasciata* Delile enhanced photosynthesis and growth in tobacco. *Mol. Biol. Rep.* 45 1745–1758. 10.1007/s11033-018-4318-4311 30159639

[B88] Yemm E. W. Cocking E. C. Ricketts R. E. (1955). The determination of amino-acids with ninhydrin. *Analyst* 80:209 10.1039/AN9558000209

