# Supplementary material for: High Photosynthetic Rates in a Solanum pennellii Chromosome 2 QTL Is Explained by Biochemical and Photochemical Changes
Source: Front Plant Sci. 2020 Jun 12;11:794. doi: 10.3389/fpls.2020.00794 (PMC7303335; doi:10.3389/fpls.2020.00794)
Supplement: Supplementary file 1 [file Presentation_1.PPTX]

## Slide 1
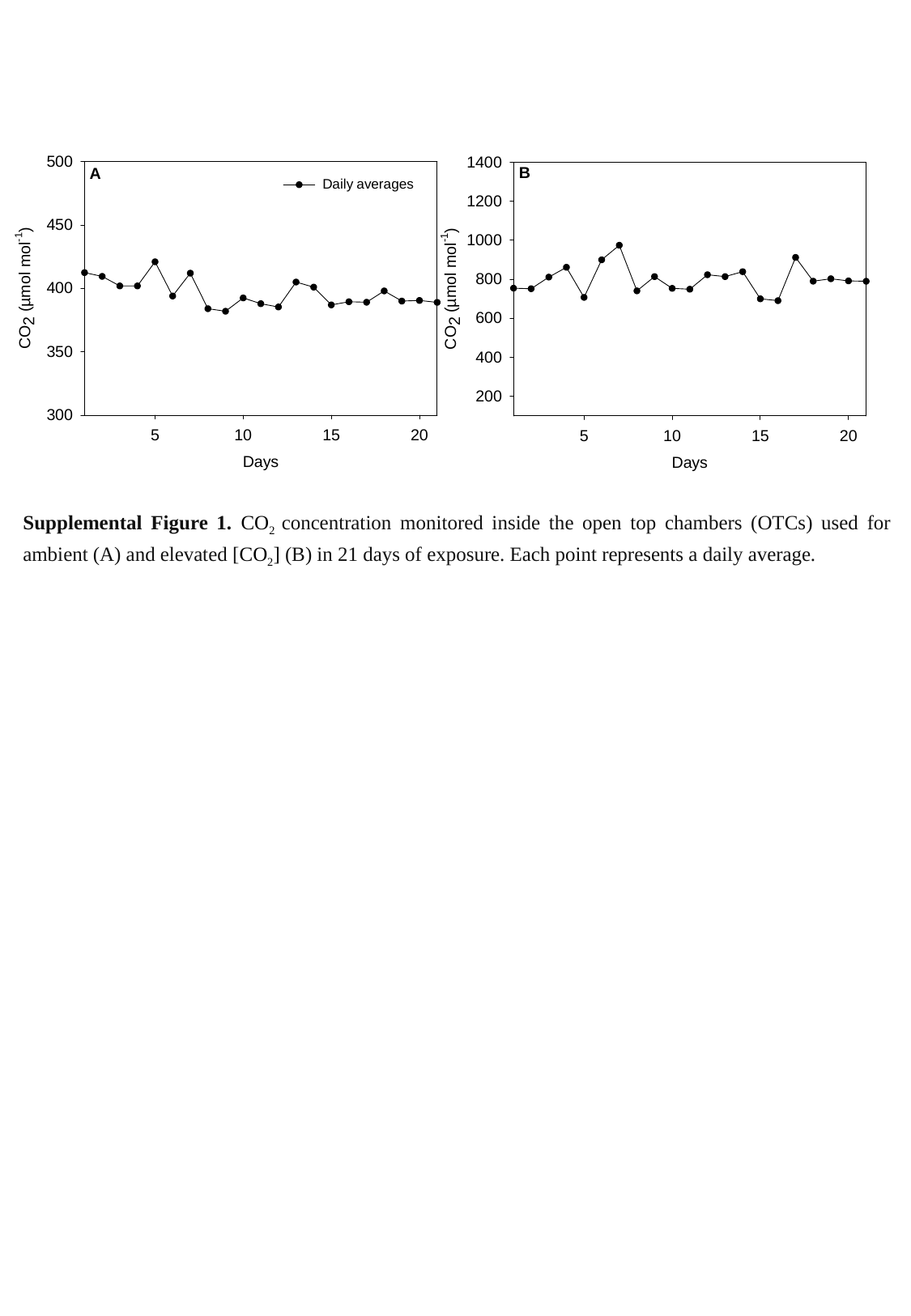

Supplemental Figure 1. CO2 concentration monitored inside the open top chambers (OTCs) used for ambient (A) and elevated [CO2] (B) in 21 days of exposure. Each point represents a daily average.
