# Supplementary material for: High Photosynthetic Rates in a Solanum pennellii Chromosome 2 QTL Is Explained by Biochemical and Photochemical Changes
Source: Front Plant Sci. 2020 Jun 12;11:794. doi: 10.3389/fpls.2020.00794 (PMC7303335; doi:10.3389/fpls.2020.00794)
Supplement: Supplementary file 2 [file Presentation_2.PPTX]

## Slide 1
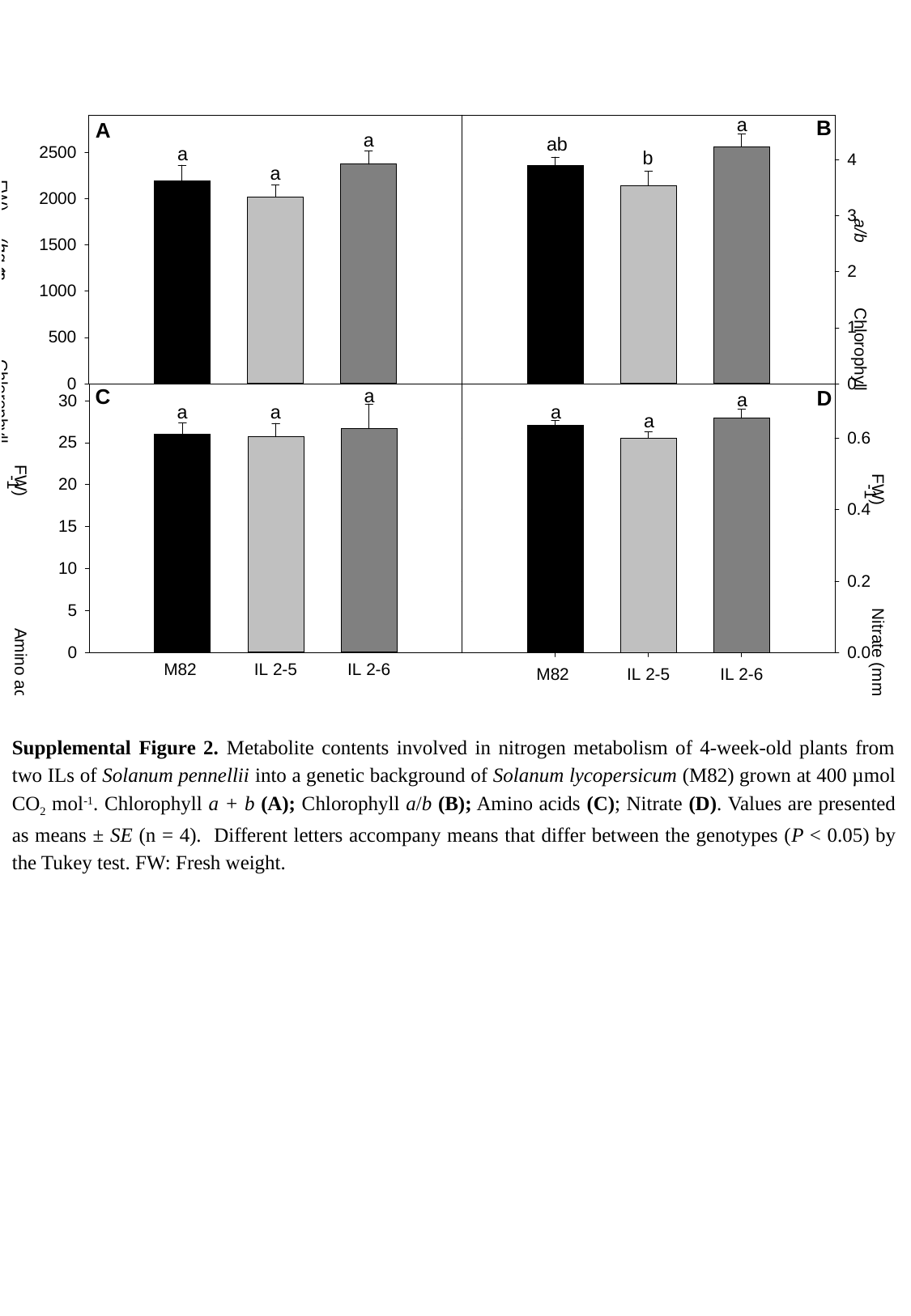

Supplemental Figure 2. Metabolite contents involved in nitrogen metabolism of 4-week-old plants from two ILs of Solanum pennellii into a genetic background of Solanum lycopersicum (M82) grown at 400 µmol CO2 mol-1. Chlorophyll a + b (A); Chlorophyll a/b (B); Amino acids (C); Nitrate (D). Values are presented as means ± SE (n = 4). Different letters accompany means that differ between the genotypes (P < 0.05) by the Tukey test. FW: Fresh weight.
