# Supplementary material for: High Photosynthetic Rates in a Solanum pennellii Chromosome 2 QTL Is Explained by Biochemical and Photochemical Changes
Source: Front Plant Sci. 2020 Jun 12;11:794. doi: 10.3389/fpls.2020.00794 (PMC7303335; doi:10.3389/fpls.2020.00794)
Supplement: Supplementary file 3 [file Presentation_3.PPTX]

## Slide 1
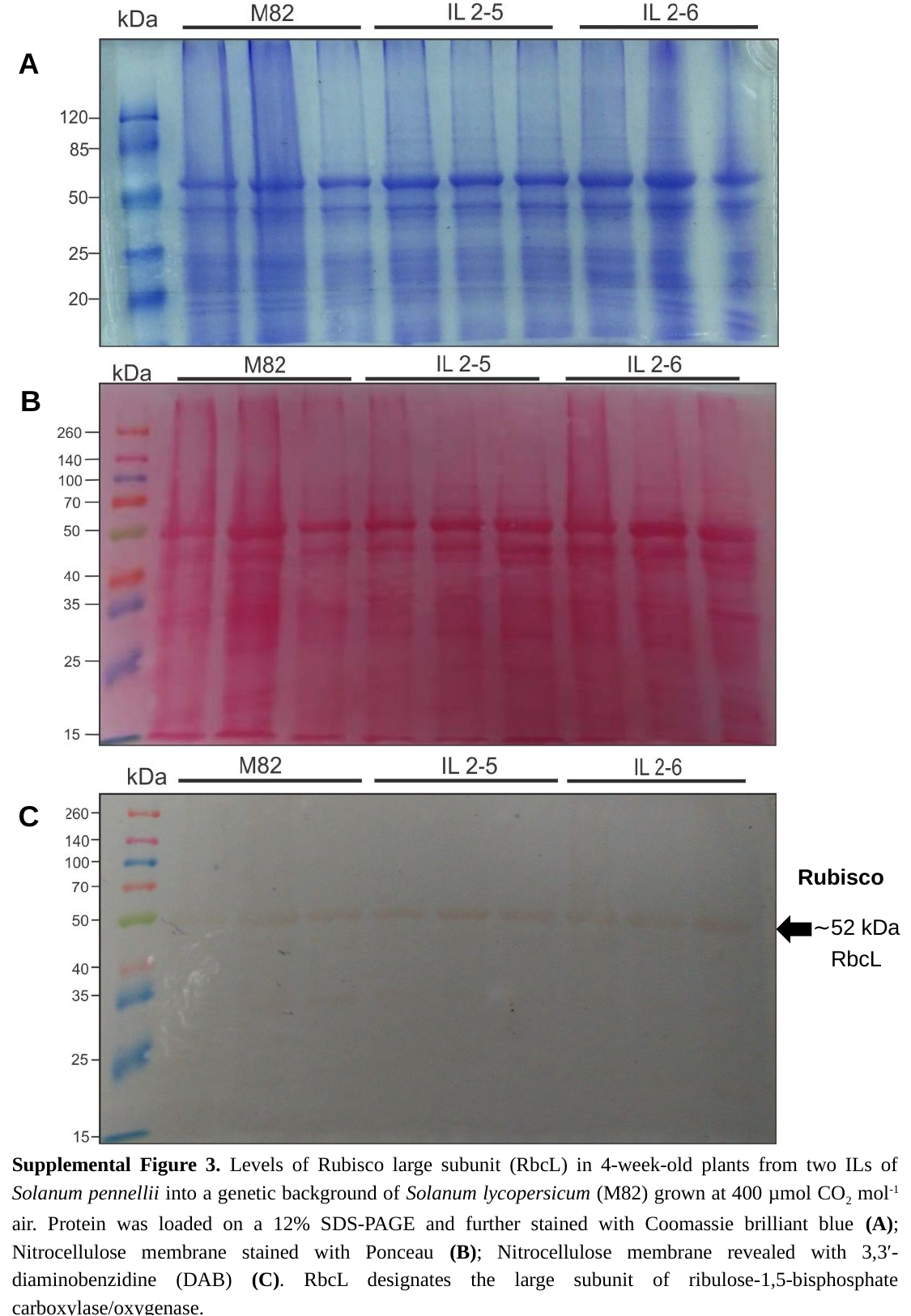

A
B
C
Rubisco
∼52 kDa
RbcL
Supplemental Figure 3. Levels of Rubisco large subunit (RbcL) in 4-week-old plants from two ILs of Solanum pennellii into a genetic background of Solanum lycopersicum (M82) grown at 400 µmol CO2 mol-1 air. Protein was loaded on a 12% SDS-PAGE and further stained with Coomassie brilliant blue (A); Nitrocellulose membrane stained with Ponceau (B); Nitrocellulose membrane revealed with 3,3′-diaminobenzidine (DAB) (C). RbcL designates the large subunit of ribulose-1,5-bisphosphate carboxylase/oxygenase.
