# Supplementary material for: High Photosynthetic Rates in a Solanum pennellii Chromosome 2 QTL Is Explained by Biochemical and Photochemical Changes
Source: Front Plant Sci. 2020 Jun 12;11:794. doi: 10.3389/fpls.2020.00794 (PMC7303335; doi:10.3389/fpls.2020.00794)
Supplement: Supplementary file 4 [file Presentation_4.PPTX]

## Slide 1
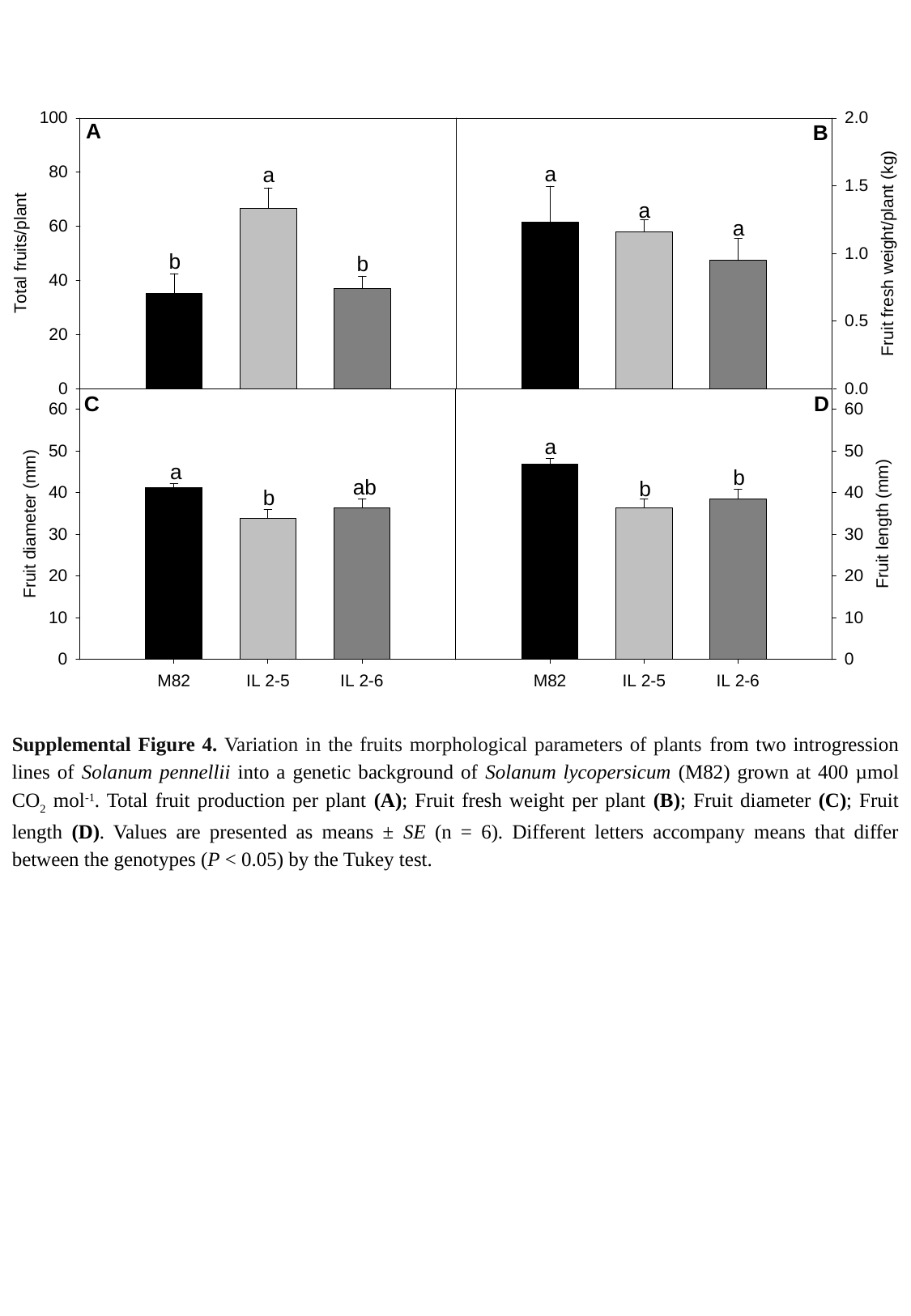

Supplemental Figure 4. Variation in the fruits morphological parameters of plants from two introgression lines of Solanum pennellii into a genetic background of Solanum lycopersicum (M82) grown at 400 µmol CO2 mol-1. Total fruit production per plant (A); Fruit fresh weight per plant (B); Fruit diameter (C); Fruit length (D). Values are presented as means ± SE (n = 6). Different letters accompany means that differ between the genotypes (P < 0.05) by the Tukey test.
