# Supplementary material for: High Photosynthetic Rates in a Solanum pennellii Chromosome 2 QTL Is Explained by Biochemical and Photochemical Changes
Source: Front Plant Sci. 2020 Jun 12;11:794. doi: 10.3389/fpls.2020.00794 (PMC7303335; doi:10.3389/fpls.2020.00794)
Supplement: Supplementary file 5 [file Presentation_5.PPTX]

## Slide 1
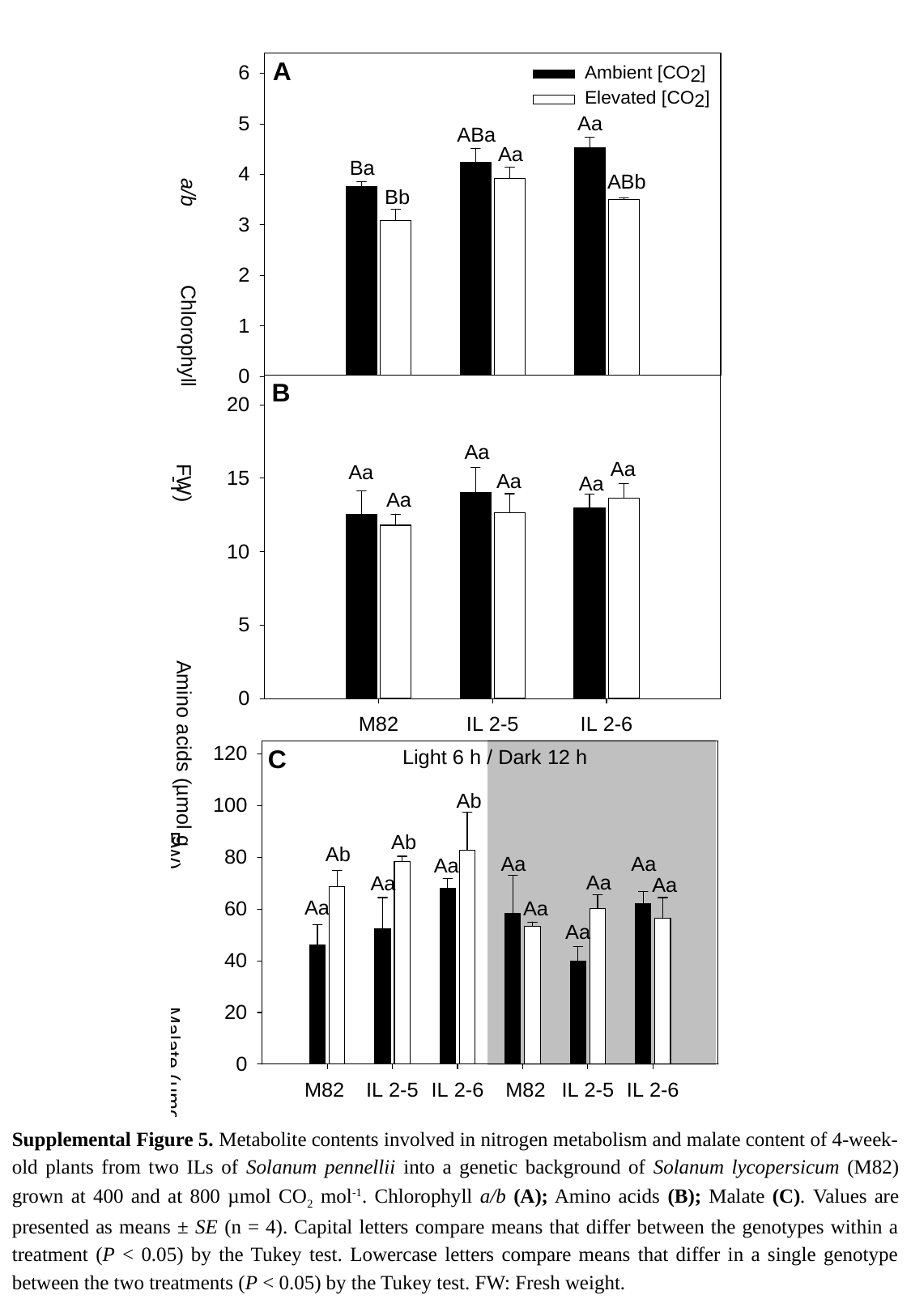

Supplemental Figure 5. Metabolite contents involved in nitrogen metabolism and malate content of 4-week-old plants from two ILs of Solanum pennellii into a genetic background of Solanum lycopersicum (M82) grown at 400 and at 800 µmol CO2 mol-1. Chlorophyll a/b (A); Amino acids (B); Malate (C). Values are presented as means ± SE (n = 4). Capital letters compare means that differ between the genotypes within a treatment (P < 0.05) by the Tukey test. Lowercase letters compare means that differ in a single genotype between the two treatments (P < 0.05) by the Tukey test. FW: Fresh weight.
