# Supplementary material for: High Photosynthetic Rates in a Solanum pennellii Chromosome 2 QTL Is Explained by Biochemical and Photochemical Changes
Source: Front Plant Sci. 2020 Jun 12;11:794. doi: 10.3389/fpls.2020.00794 (PMC7303335; doi:10.3389/fpls.2020.00794)
Supplement: Supplementary file 6 [file Presentation_6.PPTX]

## Slide 1
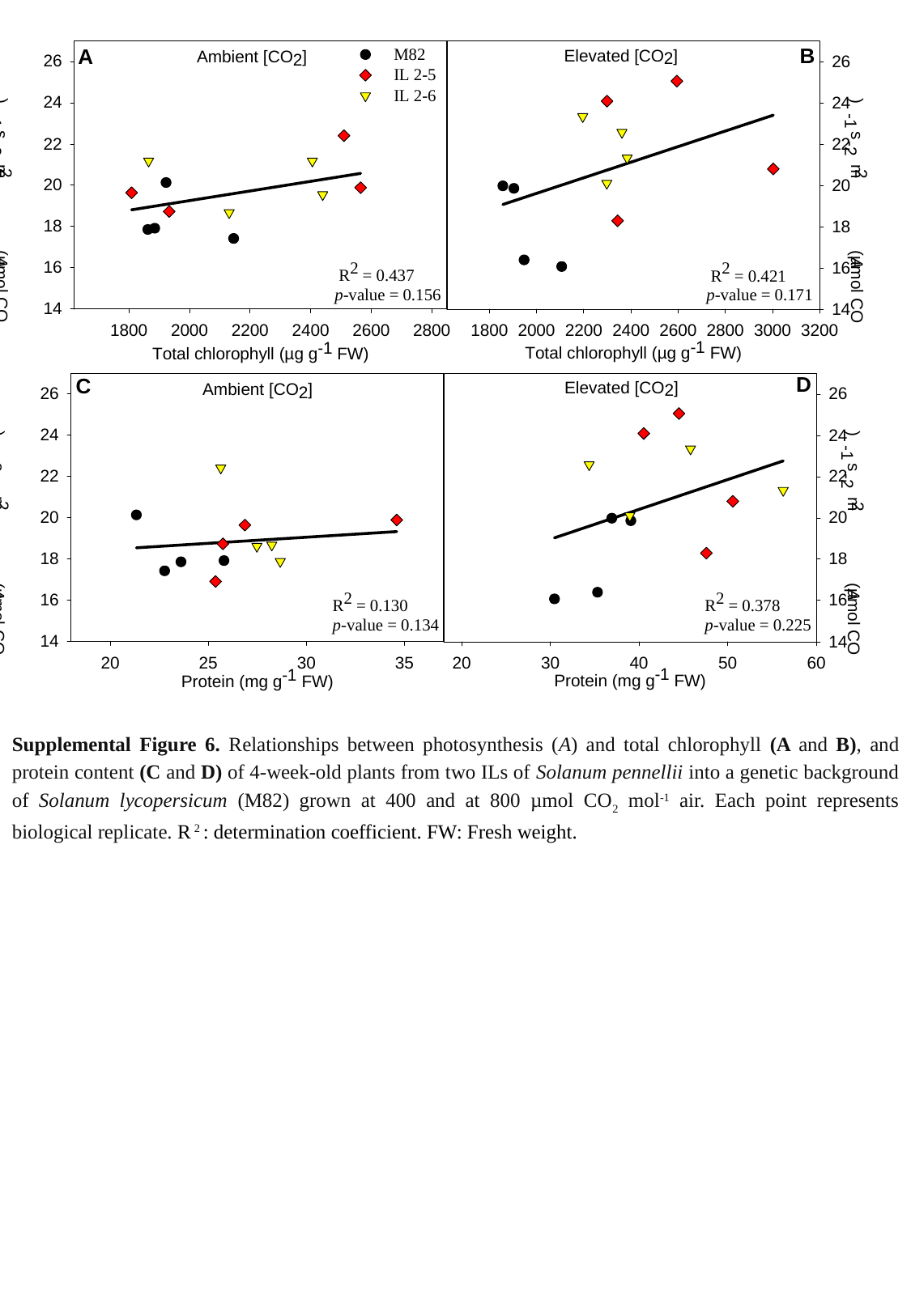

Supplemental Figure 6. Relationships between photosynthesis (A) and total chlorophyll (A and B), and protein content (C and D) of 4-week-old plants from two ILs of Solanum pennellii into a genetic background of Solanum lycopersicum (M82) grown at 400 and at 800 µmol CO2 mol-1 air. Each point represents biological replicate. R 2 : determination coefficient. FW: Fresh weight.
