# Supplementary material for: High Photosynthetic Rates in a Solanum pennellii Chromosome 2 QTL Is Explained by Biochemical and Photochemical Changes
Source: Front Plant Sci. 2020 Jun 12;11:794. doi: 10.3389/fpls.2020.00794 (PMC7303335; doi:10.3389/fpls.2020.00794)
Supplement: Supplementary file 7 [file Table_1.docx]

**Supplemental Table 1.** Photosynthetic parameters derived from light response curve of 4‐week‐old plants from two ILs of *Solanum pennellii* into a genetic background of *Solanum lycopersicum* (M82) grown at 400 µmol CO_2_ mol^-1^ air. Measurements were performed in the second terminal leaflet of the third fully expanded leaf from ILs and M82 plants.Values are presented as means ± *SE* (n = 4). Different letters accompany means that differ between the genotypes (*P* < 0.05) by the Tukey test.

| **Parameters** | | **M82** | | **IL 2-5** | **IL 2-6** |
| --- | --- | --- | --- | --- | --- |
| *A*_max_ (µmol CO_2_ m^-2^ s^-1^) | 22.39 ± 0.79 **a** | | 23.96 ± 0.72 **a** | | 23.54 ± 0.97 **a** |
| 1/ϕ (mol photons / mol CO_2_) | 14.24 ± 0.43 **b** | | 16.42 ± 0.74 **a** | | 14.61 ± 0.50 **ab** |
| LCP (µmol m^-2^ s^-1^) | 39.68 ± 1.64 **a** | | 45.60 ± 3.04 **a** | | 43.54 ± 2.92 **a** |
| LSP (µmol m^-2^ s^-1^) | 699.52 ± 20.55 **a** | | 646.41 ± 29.57 **a** | | 731.40 ± 38.02 **a** |

*A*_max_, maximum photosynthetic rate; 1/ϕ inverse of apparent quantum yield; LCP, light compensation point; LSP, light saturation point.
